# *PRDM9* losses in vertebrates are coupled to those of paralogs *ZCWPW1* and *ZCWPW2*

**DOI:** 10.1073/pnas.2114401119

**Published:** 2022-02-25

**Authors:** Maria Izabel A. Cavassim, Zachary Baker, Carla Hoge, Mikkel H. Schierup, Molly Schumer, Molly Przeworski

**Affiliations:** ^a^Bioinformatics Research Centre, Aarhus University, Aarhus 8000, Denmark;; ^b^Department of Biological Sciences, Columbia University, New York, NY 10027;; ^c^Department of Biology, Stanford University, Stanford, CA 94305;; ^d^Department of Systems Biology, Columbia University, New York, NY 10027

**Keywords:** PRDM9 evolution, genetics, recombination, comparative genomics, phylogenetics

## Abstract

In most mammals and likely throughout vertebrates, the gene *PRDM9* specifies the locations of meiotic double strand breaks; in mice and humans at least, it also aids in their repair. For both roles, many of the molecular partners remain unknown. Here, we take a phylogenetic approach to identify genes that may be interacting with PRDM9 by leveraging the fact that *PRDM9* arose before the origin of vertebrates but was lost many times, either partially or entirely—and with it, its role in recombination. As a first step, we characterize PRDM9 domain composition across 446 vertebrate species, inferring at least 13 independent losses. We then use the interdigitation of *PRDM9* orthologs across vertebrates to test whether it coevolved with any of 241 candidate genes coexpressed with PRDM9 in mice or associated with recombination phenotypes in mammals. Accounting for the phylogenetic relationship among a subsample of 189 species, we find two genes whose presence and absence is unexpectedly coincident with that of *PRDM9*: *ZCWPW1*, which was recently shown to facilitate double strand break repair, and its paralog *ZCWPW2*, as well as, more tentatively, *TEX15* and *FBXO47*. *ZCWPW2* is expected to be recruited to sites of PRDM9 binding; its tight coevolution with *PRDM9* across vertebrates suggests that it is a key interactor within mammals and beyond, with a role either in recruiting the recombination machinery or in double strand break repair.

## Significance

We take a phylogenetic approach to search for molecular partners of PRDM9, a key meiotic recombination gene, by leveraging the fact that the complete *PRDM9* gene has been lost at least 13 times independently in vertebrates. We identify two genes, *ZCWPW1* and its paralog *ZCWPW2*, whose presence or absence across vertebrates is coupled to that of *PRDM9*. ZCWPW1 was recently shown to be recruited to sites of PRDM9 binding and to aid in the repair of double strand breaks. ZCWPW2 is likely recruited to sites of PRDM9 binding as well; its tight coevolution with *PRDM9* across vertebrates suggests that it too plays an important role in mammals and beyond, either in double strand break formation or repair.

Meiotic recombination is initiated by the deliberate infliction of numerous double strand breaks (DSBs) in the genome, the repair of which yields crossover and noncrossover resolutions (reviewed in ref. 1). In mice and humans, and probably in most mammals, the localization of almost all DSBs is specified through the binding of PRDM9 (2–4). Yet the presence of a PRDM9 binding site is far from sufficient for a DSB to be made; a number of additional factors modulate whether PRDM9 binds or act downstream of PRDM9 binding (5–7).

The mechanism by which PRDM9 directs recombination to the genome is partially understood; it binds DNA through a C2H2 zinc finger (ZF) array and contains a SET domain that trimethylates histones H3K4 and H3K36 (8, 9). These epigenetic marks together recruit the DSB machinery, notably SPO11 (which makes the DSBs), through intermediates that remain unknown (10). In addition to the ZF binding array and SET domain, most mammalian *PRDM9* genes also have two other domains, KRAB and SSXRD, whose functions are unclear.

The complete PRDM9 protein, with all four domains, originated before the diversification of vertebrates, so has been conserved for hundreds of millions of years (11, 12). Yet the entire gene has also been lost numerous times, including in birds and canids (13–15). In these species, recombination occurs preferentially around promoter-like features, notably 5′-C-phosphate-G-3′ (CpG) islands (11, 15–17). A possible explanation is that in the absence of the histone marks laid down by PRDM9, the recombination machinery defaults to those residual H3K4me3 marks found in the genome, often associated with sites of transcription initiation, or perhaps simply to wherever DNA is accessible (15, 18). The same concentration of DSBs around promoter-like features is seen in *Prdm9*^−/−^ mice (18) and in a woman who carries two loss of function copies of *PRDM9* identical by descent (19). These findings suggest that mammals that carry an intact *PRDM9* retain the mechanism to direct recombination employed by species lacking *PRDM9*, but it is normally outcompeted by PRDM9 binding.

In addition to complete losses of *PRDM9*, multiple partial losses have occurred independently (e.g., in platypus and various fish lineages), usually involving the truncation of the N-terminal KRAB and SSXRD domains (11). Although these partial *PRDM9* orthologs evolve under selective constraint and thus must have some conserved function (11), several lines of evidence indicate that they do not direct recombination to the genome. For one, only in species with a complete *PRDM9* is the ZF unusually rapidly evolving in its binding affinity (11). Since the rapid evolution of the ZF is thought to arise from the role of PRDM9 in recombination (3, 20, 21), this evolutionary pattern suggests that all four domains are required for DSB localization. Empirical data support this notion: In swordtail fish carrying one *PRDM9* ortholog that lacks the KRAB and SSXRD domains as well as in a mouse model in which only the KRAB domain is knocked out, recombination events are concentrated at promoter-like features, as in species lacking *PRDM9* altogether (11, 22). Therefore, the KRAB domain at least appears to be necessary for PRDM9 to direct recombination, likely by mediating interactions with other proteins (22, 23).

Conversely, the presence of a complete *PRDM9* with a rapidly evolving ZF outside of mammals (11) suggests that PRDM9 also directs recombination to the genome in these species, as has been reported for rattlesnakes (24). Thus, at least two mechanisms for directing meiotic recombination are interdigitated within mammals as well as seemingly throughout the vertebrate phylogeny.

In addition to specifying the locations of DSBs, PRDM9 has recently been discovered to play a second role, in the downstream repair of DSBs (25–27). In mice and humans, DSBs at which PRDM9 is bound on both homologs are more likely to be efficiently repaired and to result in a crossover; in contrast, DSBs at which PRDM9 is only bound on one of the two homologs are delayed in their repair (27, 28). If these “asymmetric” DSBs are overwhelming in number—as is the case in certain hybrid crosses in mice—this delay can lead to asynapsis and infertility (29, 30).

Although the role of PRDM9 in DSB repair is still poorly understood, recent papers report that it is facilitated by ZCWPW1, which binds H3K4me3 and H3K36me3 (25–27) and is expressed alongside PRDM9 in single-cell data from mouse testes (31). One line of evidence that led to the discovery of *ZCWPW1* is its phylogenetic distribution; although it too has been lost numerous times in vertebrates, it is found in seven clades that carry an intact *PRDM9* (26, 27). Thus, while the distribution of the two genes across species is not perfectly concordant, it is strongly suggestive of coevolution.

The example of *ZCWPW1* highlights the potential power of coevolutionary tests to identify additional molecular partners of PRDM9. While not all partners of PRDM9 will coevolve with it, genes whose losses are coupled to those of PRDM9 are strong candidates for molecular interactors. We therefore applied this approach systematically; we considered a set of 241 candidate genes that are known to be involved in recombination in model organisms (32), associated with recombination phenotypes in a human genome-wide association study (33), or coexpressed with PRDM9 in single-cell data from mouse testes (31) and tested for their cooccurrence with *PRDM9* across 189 vertebrate species. After verifying our initial gene status calls in whole-genome data and, for a subset of species, with RNA-seq data, we identified the paralog of *ZCWPW1*, *ZCWPW2*, as coevolving with *PRDM9* and found more tentative evidence for two additional genes, *TEX15* and *FBXO47*.

## Results

### A Revised Phylogeny of PRDM9.

We previously reported that the complete *PRDM9* gene, including the KRAB, SSXRD, and SET domains, arose before the origin of vertebrates and was lost independently a number of times, both in its entirety and partially (through the loss of its N-terminal domains; Ref. 11). Here, we leverage the independent losses of *PRDM9* in order to identify genes that are coevolving with *PRDM9*—specifically, that tend to be present in the same species as *PRDM9* and lost (partially or entirely) when *PRDM9* is no longer complete.

As a first step, we characterized the phylogenetic distribution of *PRDM9* in light of new genome sequences published since our initial analysis (11). To this end, we created a curated dataset of 747 vertebrate *PRDM9* sequences by analyzing publicly available protein sequences from RefSeq (34), whole-genome sequences, and RNA-seq data from testes samples, as well as four RNA-seq datasets from testes samples that we generated (see *Methods*; *SI Appendix*, Fig. S1 and Tables S1–S3). For this analysis, we defined *PRDM9* orthologs as complete if they contain both the KRAB and SET domains; we did not consider the SSXRD domain because its short length makes its detection at a given e-value threshold unreliable nor the ZF array because its repetitive structure makes it difficult to sequence and assemble reliably.

**Fig. 1.**
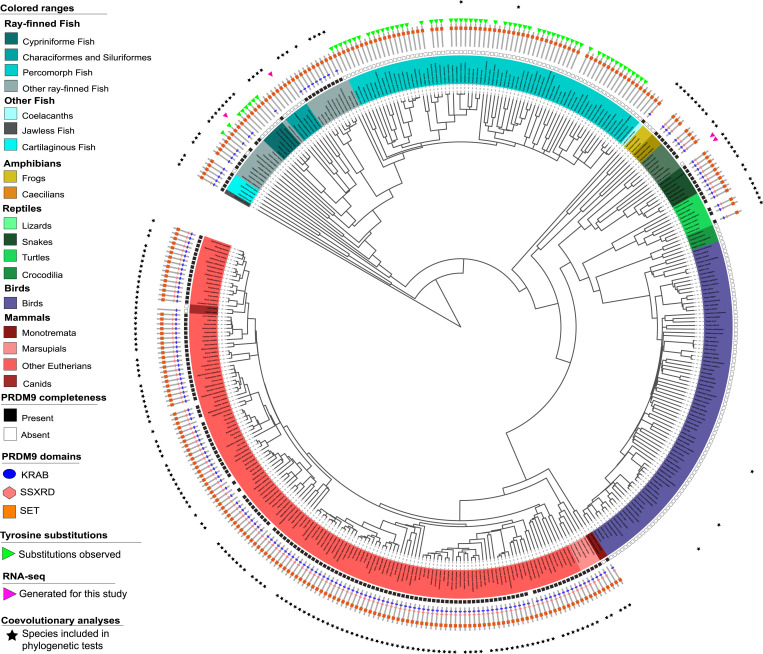
The phylogenetic distribution of PRDM9 and its domain architecture across vertebrates. The inferred *PRDM9* status of 432 vertebrate species is shown. Branch lengths were computed based on the TimeTree database. For 28 species not present in the database, we used branch length information from a close evolutionary relative; for 14 species in which we made PRDM9 calls, we were unable to find such a substitute, so they are not represented. Different vertebrate clades are indicated by colored segments, with salmon for mammals, cyan for fish, mustard for amphibians, green for reptiles, and purple for birds. In the inner circle, squares indicate whether *PRDM9* is complete (solid black) or incomplete/absent (open black); for species with an uncertain *PRDM9* status, no box is shown. The PRDM9 domain architecture of each species is shown with a cartoon, in which the presence of a KRAB domain is indicated in blue, SSXRD in pink, and the SET domain in orange. Green triangles indicate species that only carry *PRDM9* orthologs with substitutions at putatively important catalytic residues in the SET domain (see *SI Appendix*, Table S4). The tree was drawn using itool (https://itol.embl.de/); an interactive version is available at https://itol.embl.de/shared/izabelcavassim.

Across 446 species, we identified 221 species with at least one complete *PRDM9* ortholog and 225 species without a complete *PRDM9* ortholog (Fig. 1; *SI Appendix*, Table S4). Notably, we were able to uncover complete *PRDM9* orthologs in a number of species for which we had previously predicted partial or complete losses (11), including in the Tasmanian devil (*Sarcophilus harrisii*), the Atlantic cod (*Gadus morhua*), and the Atlantic herring (*Clupea harengus*), as well as in a handful of eutherian mammals that we had previously only investigated using RefSeq (*SI Appendix*, Table S4). We also found a complete *PRDM9* ortholog in caecilians and in two species of frogs, suggesting that the previously reported loss of *PRDM9* in amphibians (11) reflects at least one loss in salamanders and more than one independent loss in frogs. We note, finally, that by the approach taken here, the *PRDM9* ortholog from the Australian ghostshark (*Callorhinchus milii*) is considered to be complete (in contrast to in Baker et al., Ref. 11, where we also relied on the SSXRD domain; *SI Appendix*, Table S4). Confirming our earlier finding (11, 15–17), there is a near-perfect correspondence between species carrying putatively complete PRDM9 orthologs (i.e., with KRAB domains) and those for which we identify rapidly evolving PRDM9 ZF arrays (*Methods*; *SI Appendix*, Tables S1 and S4).

Based on the phylogenetic relationships among species given by the TimeTree tool (http://timetree.org/; Ref. 35), we inferred 23 putative complete or partial losses of *PRDM9* across the 446 vertebrates considered (Fig. 1; *SI Appendix*, Table S4). These putative losses include six previously reported ones (11, 13–15), each observed in two or more closely related species: in percomorph fish, cypriniformes fish, characiformes and siluriformes fish, osteoglossomorpha fish, birds and crocodiles, and canids. In turn, the putative losses of PRDM9 in polypteriformes fish, salamanders, and three clades of frog species (*Xenopus*, *Dicroglossidae*, and *Bufonidae*) were each supported by the absence of PRDM9 in the genomes of two or more closely related species. We were further able to verify the absence of PRDM9 in two *Xenopus* frogs and in two salamanders using RNA-seq data from testes: despite sufficient power to detect a set of six highly conserved meiotic genes in each species, we did not detect the expression of any complete PRDM9 orthologs (*SI Appendix*, Table S3).

We also failed to find *PRDM9* in RefSeq or the whole-genome sequence of the green anole (*Anolis carolinensis*). We verified this absence of *PRDM9* by collecting RNA-seq data from testes in the green anole as well as in the fence lizard (*Sceloporus undulatus*), for which neither a RefSeq nor a genome sequence were available at the time, and did not detect PRDM9 expression in either species (*SI Appendix*, Figs. S2 and S3 and Table S3). Given the presence of a complete *PRDM9* in bearded dragons (*Pogona vitticeps*), it appears that this loss of *PRDM9* occurred in a lineage basal to the common ancestor of green anoles and fence lizards, over 99 Mya but less than 157 Mya (*SI Appendix*, Fig. S4).

The remaining 11 putative absences of *PRDM9* are observed in single species; we were able to verify the call using testis RNA-seq data for the platypus (*Ornithorhynchus anatinus*), but not for the remaining 10 species, so their *PRDM9* status remains uncertain. Thus, in total, we identified at least 13 independent *PRDM9* losses in vertebrates, and possibly as many as 23 (Fig. 1; *SI Appendix*, Table S4). The 13 losses occur in five clades of ray-finned fish (percomoprhs, cypriniformes, characiformes and siluriformes, osteoglossomorphs, and polypteriformes), in four clades of amphibians (*Xenopus*, *Dicroglossidae*, and *Bufonidae* frogs and salamanders), in two clades of reptiles/birds (birds and crocodiles and the clade of lizards comprised of anoles and fence lizards), and in two clades of mammals (platypus and canids). At least 12 of the 13 losses are relatively old (*SI Appendix*, Fig. S5); the most recent case manifest in these data is either the one that happened in the branch leading to platypus (sometime in the last 46 Mya) or the one in canids, which could be as recent as 14.2 Mya (*SI Appendix*, Fig. S5).

### Identifying Genes Coevolving With PRDM9.

We selected 193 candidate genes based on their coexpression with PRDM9 in single-cell RNA-seq data from mouse testes (specifically, in component 5; *Methods*; Ref. 31) (*SI Appendix*, Fig. S6 *A* and *B*). To this set, we added any gene associated with variation in recombination phenotypes in humans (33) as well as genes known to have a role in mammalian meiotic recombination from functional studies (summarized in Ref. 32). Together, these three sources provided a total of 241 genes to evaluate for possible coevolution with *PRDM9* (*SI Appendix*, Table S5 and Fig. S6*C*).

We evaluated the presence or absence of these 241 genes across the NCBI RefSeq database of 189 species. These 189 species were downsampled from the larger phylogenetic tree to preserve at most three species with high quality genomes below each *PRDM9* loss, thereby minimizing phylogenetic signals driven by variation in genome quality (*Methods*; *SI Appendix*, Fig. S7). The phylogeny includes representative species for 11 of the 13 inferred PRDM9 losses (*Methods*). Species of *Bufonidae* frogs and salamanders were not included because of the absence of available gene annotations; moreover, because of the lack of gene annotations for frog species with PRDM9, within these 189 species, the losses in *Xenopus* and *Dicroglossidae* frogs cannot be distinguished from a single event.

We encoded a gene as present when it contained all the domains found in four representative vertebrates with a complete *PRDM9* and absent if it lacked one or more of those domains (*Methods*). Many of the 241 genes are present in every sampled vertebrate and hence provide no information in our coevolutionary test of presence and absence; specifically, we found apparently complete orthologs for 102 candidate genes in all 189 species used in the phylogenetic test. We therefore focused on the remaining 139 genes, each of which has been lost at least once among vertebrate species evaluated here; the matrix of 189 × 139 gene status calls is presented in *SI Appendix*, Table S6.

**Fig. 2.**
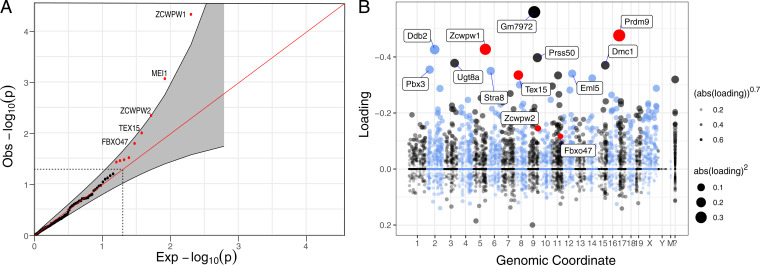
Phylogenetic tests and genes coexpressed with PRDM9 in single-cell mouse testes data. (*A*) Quantile-quantile plot of the *P* values obtained from the phylogenetic tests run on 139 genes that appeared to have been lost at least once in the 189 vertebrate species considered. Genes that are significant at the 5% level are shown in red (outside the dashed lines), and a pointwise 95% confidence interval is shown in gray. Genes with an FDR ≤ 50% are annotated. (*B*) Loadings for one of 46 components (component 5) inferred from single-cell–expression data in mouse testes (31), in which PRDM9 is most highly expressed. The dot sizes are proportional to the square of the absolute value of the loading. *PRDM9* and the three genes identified in our phylogenetic tests with *P* < 0.05 are shown in red. Mouse genomic coordinates are displayed. Panel B was made from summary statistics provided by ref. 31, using SDAtools (https://github.com/marchinilab/SDAtools/).

We tested for the coevolution of *PRDM9* and each candidate gene by comparing a null model with independent rates of gains and losses of *PRDM9* and of the focal gene to an alternative model in which the state transition rates of the two genes are dependent on one another, using the maximum likelihood approach within *BayestraitsV3* (36, 37) (*SI Appendix*, Table S7 and Fig. S8). By this approach, we identified nine significant hits at the 5% level (uncorrected for multiple tests): in order of increasing *P* values, *ZCWPW1*, *MEI1*, *ZCWPW2*, *TEX15*, *FBXO47*, *ANKRD31*, *NFKBIL1*, *SYCE1*, and *FMR1NB*. We focused on the top five, for which the false discovery rate (FDR) value is below 50% (Table 1; Fig. 2*A*).

**Table 1.** Results of phylogenetic tests

Gene	Start position	Gene source	LogLik H0	LogLik Ha	*P* value	FDR	*P* value for improved status calls
ZCWPW1	chr7:100400826	1	−65.941	−53.35647	4.651e−05	0.0064	1.948e−03
MEI1	chr22:41699503	3,1	−54.200	−44.779	8.442e−04	0.0586	NA
ZCWPW2	chr3:28348721	1	−67.711	−60.146	4.437e−03	0.2055	5.171e−06
TEX15	chr8:30831544	1	−138.430	−131.764	9.760e−03	0.3391	8.682e−02
FBXO47	chr17:38936278	2	−53.678	−47.559	1.566e−02	0.4354	1.566e−02

We focused on the five genes that had an FDR ≤ 50%, improved the ortholog status calls, and reran the phylogenetic tests for four of them (all but *MEI1*, which turned out to be present in all species considered; see Methods). Gene source refers to the criterion by which the gene was originally included among our lists of candidates: 1) It is coexpressed with PRDM9 in single-cell mouse testes data (31), or 2) variants assigned to the gene are associated with variation in recombination phenotypes in humans (33), or 3) the gene was previously known to have a role in mammalian meiotic recombination from functional studies (32) (see *Methods*). Start positions are based on the human reference genome GRCh38/hg38. NA, not applicable.

**Fig. 3.**
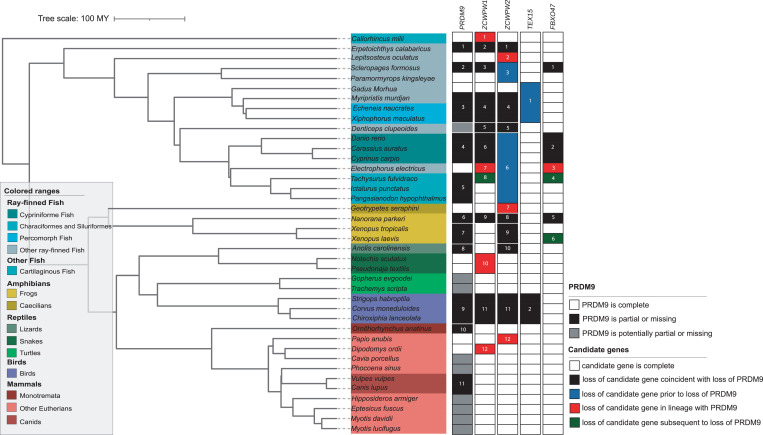
A summary of the phylogenetic distribution of *PRDM9* and the four candidate genes across 189 species. Ortholog calls for candidate genes were based on a search of gene models within whole-genome sequences (*Methods*), and the phylogenetic test for coevolution with *PRDM9* was rerun on these updated calls; updated *P* values for the phylogenetic test are shown in Table 1. Solid white and black rectangles indicate whether *PRDM9* is present or absent, respectively, and gray rectangles lineages for which the status of *PRDM9* is uncertain (*Methods*). For candidate genes, white rectangles are instances in which the gene is present and complete and solid black rectangles indicate when loss of candidate gene is coincident with that of *PRDM9*. Solid blue rectangles point to instances in which loss of candidate gene occurred prior to that of *PRDM9* and green rectangles when it occurred subsequent to that of *PRDM9*. Red rectangles denote cases in which loss of the candidate gene occurred in lineages with a complete *PRDM9*. The full phylogenetic distribution of *PRDM9* and candidate genes is in *SI Appendix*, Fig. S10.

We sought to verify the phylogenetic distribution of the top genes by developing curated datasets of high confidence orthologs, as we had for *PRDM9* (*Methods*; Fig. 3; *SI Appendix*, Tables S1 and S8 and Figs. S9 and S10). In doing so, we were able to identify *MEI1* orthologs from the whole-genome assemblies of each species missing *MEI1* in our initial dataset, resulting in the presence of *MEI1* in every species considered (*SI Appendix*, Table S1); thus, it appears that its inferred coevolution with *PRDM9* based on RefSeq calls is artifactual (*Methods*). Rerunning the phylogenetic test on the curated ortholog sets for the remaining four genes, *TEX15* is no longer significant at the 5% level (*P* = 0.086), possibly because the curation uncovered an intact *TEX15* ortholog in anoles. *ZCWPW1* and *ZCWPW2* are still highly significant; for *FBXO47*, the curation did not reveal any discrepancy with the initial calls, so the *P* value remains the same.

Our approach therefore uncovered two genes with clear-cut evidence of coevolution with *PRDM9*, the paralogs *ZCWPW1* and *ZCWPW2*, and more tentative support for two others, *TEX15* and *FBX047*. *ZCWPW1*, *ZCWPW2*, and *TEX15* were among our initial list of 241 candidate genes because they are coexpressed with PRDM9 in single-cell testis data from mouse (Fig. 2*B*; *SI Appendix*, Fig. S6) (31). *FBX047* was not included by that criterion but because missense mutations in the gene are associated with variation in the total genetic map length in humans in both sexes (33). In mice, the expression of *FBXO47* is testis-specific (38), and the gene is expressed in the component in which *PRDM9* had the highest loading (albeit with a smaller loading; Ref. 31; Fig. 2*B*; see also Ref. 39).

Like *PRDM9*, *ZCWPW1*, *ZCWPW2*, *FBXO47*, and *TEX15* are inferred to have been present in the common ancestor of vertebrates. In the following sections, we describe the distribution of each of the four genes across the phylogeny of 189 species and the patterns that give rise to the evidence of statistical association with *PRDM9*—in particular, the correspondence between their distributions and that of 11 well-supported losses of *PRDM9*, as well as of nine species for which the status of *PRDM9* is uncertain. Moreover, in lineages in which *ZCWPW1*, *ZCWPW2*, *FBX047*, or *TEX15* are present despite the absence of *PRDM9*, we evaluate if the genes appear to be under relaxed selective constraint, by testing whether ω = dn/ds is higher in lineages without a complete *PRDM9* than in those that still carry PRDM9 (where dn is the rate of nonsynonymous substitutions and ds the rate of synonymous substitutions; see *Methods*).

### *ZCWPW1* and *PRDM9* Coevolution.

Our finding that *ZCWPW1* is coevolving with *PRDM9* (*P* = 0.0019 in the curated set; Table 1) is in line with previous reports of an association in vertebrates between the presence and absence of *ZCWPW1* and *PRDM9* orthologs (26, 27). Here, we found an even tighter coupling of *PRDM9* and *ZCWPW1* than previously documented. Specifically, we inferred 12 losses of *ZCWPW1* among 189 species used in our phylogenetic test, distributed across 17 species that lack *ZCWPW1* entirely and two species carrying partial *ZCWPW1* genes (with the PWWP domain but not the zf-CW domain; *SI Appendix*, Table S8). Because of its known relationship with PRDM9, *ZCWPW1* can be viewed as a positive control for our approach.

Seven of the *ZCWPW1* losses occur among the 11 well-supported losses of *PRDM9*: in cypriniformes fish, percomorph fish (*Euacanthomorphacea*), siluriformes fish, polypteriformes fish, osteoglossomorpha fish, birds, and *Dicroglossidae* frogs. An additional *ZCWPW1* loss occurred in the denticle herring (*Denticeps clupeoides*), a species for which the status of *PRDM9* is uncertain. The remaining four losses of *ZCWPW1* seem to break the pattern in that they occur in lineages containing a complete *PRDM9* gene. However, three are observed only in a single species and may be spurious. Therefore, across the tree, there is only one well-supported case of a taxon with an intact *PRDM9* that has nonetheless lost *ZCWPW1*, supported by two closely related species, the tiger snake (*Notechis scutalus*) and the eastern brown snake (*Pseudonaja textilis*) (*SI Appendix*, Table S8).

Inversely, *ZCWPW1* has been retained in several lineages in which the absence of PRDM9 is well supported: in two siluriformes fish, two frogs, the green anole, platypus and canids. Moreover, there is no evidence for a relaxation of selection in these lineages (*P* > 0.13; *SI Appendix*, Table S9), with the intriguing exception of platypus (*P* = 0.038; *SI Appendix*, Table S9).

In mice as well as human cell lines, ZCWPW1 binds two marks laid down by PRDM9: The zf-CW domain binds H3K4me3 and the PWWP domain H3K36me3 (25–27). Thus, the coevolution across vertebrates likely reflects a conserved molecular interaction between ZCWPW1 and PRDM9 as reader and writer of these dual histone modifications, both within mammals and beyond.

### *ZCWPW2* Also Coevolves With *PRDM9*.

Intriguingly, the strongest association with the presence or absence of *PRDM9* is that of the paralog of *ZCWPW1*, *ZCWPW2* (*P* = 5 × 10^−6^; Table 1). Among the 189 species, there are 12 independent losses, distributed across 21 species that appear to lack *ZCWPW2* altogether and three that contain partial *ZCWPW2* genes (two with the PWWP domain but not the zf-CW domain and one with the reverse; *SI Appendix*, Table S8).

Six of the *ZCWPW2* losses occur among the 11 well-supported losses of an intact *PRDM9*: in percomorph fish, polypteriformes fish, *Xenopus* frogs, *Dicroglossidae* frogs, birds, and the green anole. In order to distinguish whether the absence of *ZCWPW2* in *Xenopus* and *Dicroglossidae* frogs reflects a single loss or multiple events, we investigated the status of *ZCWPW2* in an additional species of frog with *PRDM9* (*Ranitomeya imitator*). We were able to successfully identify a complete *ZCWPW2* ortholog in this species, suggesting that *ZCWPW2* has indeed been lost at least twice within frogs, possibly coincident with *PRDM9* in each case. *ZCWPW2* is also absent in a clade encompassing cypriniformes fish and siluriformes fish, as well as the electric eel (*Electrophorus electricus*), which has an intact *PRDM9*. This phylogenetic distribution suggests that the loss of *ZCWPW2* may have occurred before the losses of *PRDM9* in both cypriniformes fish and siluriformes fish. Also suggestive of this order of loss, *ZCWPW2* is absent in osteoglossomorpha fish (the Asian arowana, *Scleropages formosus*); in this case, the gene is also absent from the closest evolutionary relative in the tree, the elephantfish (*Paramormyrops kingsleyae*), which carries *PRDM9*.

Among the nine species for which the status of *PRDM9* is uncertain, *ZCWPW2* is absent in the denticle herring (*D. clupeoides*). The remaining three cases of *ZCWPW2* loss are each observed in a single species carrying an intact PRDM9, without supporting lines of evidence.

In summary, in the few cases with *PRDM9* but not *ZCWPW2*, we cannot verify the loss of *ZCWPW2*; conversely, the only species with *ZCWPW2* but that clearly lack *PRDM9* are canids and the platypus, the two lineages that experienced the most recent losses of *PRDM9* (*SI Appendix*, Table S8).

This observation suggests that the retention of *ZCWPW2* in the two mammalian lineages that lack *PRDM9* could simply be a lag. Consistent with this hypothesis, there is statistical support for a relaxation of constraint on *ZCWPW2* in lineages that lack PRDM9 (*P* = 0.0003; *SI Appendix*, Table S9) and in canid lineages, *ZCWPW2* is no longer under any discernible selective constraint (testing against a model of ω = 1, *P* = 0.307; *SI Appendix*, Table S9).

**Fig. 4.**
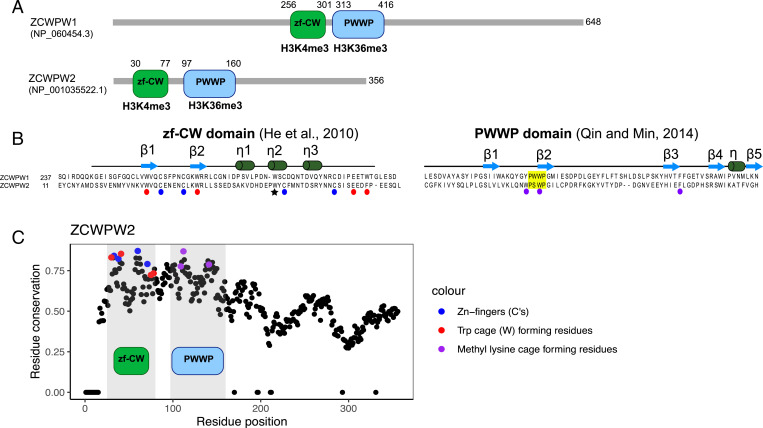
Domain architecture *ZCWPW1* and *ZCWPW2*. (*A*) Amino acid sequence and domain structure composition of genes *ZCWPW1* and *ZCWPW2* in humans. (*B*) The ZF-CW domain structure includes the fingers (residues indicated by blue circles) and an aromatic cage (red) expected to bind to H3K4me3 (61), and the star indicates the third Trp residue that is thought to stabilize the fold by hydrophobic interactions (61). The PWWP domain (yellow) is expected to bind to histone H3K36me3 through a hydrophobic cavity composed of three aromatic residues (purple) (62). The secondary structures of zf-CW and PWWP domains are represented above sequences. (*C*) Conservation of residues in *ZCWPW2* across vertebrates, with those residues recognizing modifications on the histone tail colored in blue, red and purple. Positions in the *ZCWPW2* alignment with >30% of gaps were ignored, and the conservation score was set to 0. A similar plot for the conservation of residues in *ZCWPW1* was previously reported (figure 1B in Ref. 27).

Like its paralog, ZCWPW2 contains zf-CW and PWWP domains, predicted to bind H3K4me3 and H3K36me3, respectively (Fig. 4*A*; *SI Appendix*, Fig. S11). As in ZCWPW1 (26, 27), these domains are highly conserved, especially at residues with predicted binding properties (Fig. 4 *B* and *C*), suggesting that ZCWPW2 is also recruited to sites of PRDM9 binding.

### The Distribution of *FBXO47* and *TEX15* Orthologs.

We identified two additional genes, *FBX047* and *TEX15*, that may be coevolving with *PRDM9*, with *P =* 0.016 and *P* = 0.087 based on the curated calls, respectively (Table 1). TEX15 is coexpressed with PRDM9 in two components inferred from single-cell data from mice, active during preleptotene and zygotene (*SI Appendix*, Fig. S6). The statistical evidence for coevolution stems from the fact that *TEX15* is missing in two taxa lacking *PRDM9*: birds and percomorph fish. *TEX15* is also absent in the Atlantic cod (*G. morhua*), suggesting that the loss of *TEX15* that led to its absence in percomorph fish occurred before that of *PRDM9*. All of the other 189 species considered have an intact *TEX15* (*SI Appendix*, Table S8). Among lineages where *PRDM9* is absent or incomplete, there is evidence for relaxed constraint on TEX15 compared to lineages with an intact PRDM9 (*P* = 0.0036 for fish and *P* = 0.015 for mammals), but ω remains significantly below 1 (*SI Appendix*, Table S9).

The statistical evidence for coevolution is a bit stronger for *FBX047*, which has been lost five times in groups in which *PRDM9* is absent: in cypriniformes fish, osteoglossomorpha fish, siluriformes fish, and in *Xenopus* and *Bufonidae* frogs. In fish, ω is higher when *PRDM9* is absent or incomplete than in species in which it is intact (*P* = 0.0023), but there is still evidence of selection constraint on *FBX047* (i.e., ω < 1); in mammals, there is no evidence for relaxation of constraint on *FBX047* in species without an intact PRDM9 (*SI Appendix*, Table S9).

Intriguingly, *FBXO47* is additionally absent in the electric eel, a species that carries a complete *PRDM9* gene but lacks both *ZCWPW1* and *ZCWPW2*. More generally, testing for the coevolution of the candidate genes with each other, a null model in which the state transitions of *FBXO47*, *ZCWPW1*, and *ZCWPW2* are independent is rejected for all pairs of genes (maximal *P* < 6 × 10^−3^; *SI Appendix*, Table S10*A*), and *P* values are lower for *FBXO47* and *ZCWPW1*, or *FBXO47* and *ZCWPW2*, than for *FBXO47* and *PRDM9*.

## Discussion

### Summary.

By extending the reconstruction of *PRDM9* to 446 vertebrate species, we identified 13 losses that are supported by more than one species or by independent evidence, and possibly as many as 23. Focusing on a subset of 189 species that capture 11 state transitions of *PRDM9*, we tested whether *PRDM9* transitions coincide with those of 139 candidate genes lost at least once across vertebrates. After carefully vetting the ortholog calls for our top five signals, we identified two genes that are clearly coevolving in their presence and absence with *PRDM9*, *ZCWPW1* and its paralog *ZCWPW2*, and two for which the evidence is weaker, *FBXO47* and, most tentatively, *TEX15*.

### Dual Roles of PRDM9 Across Vertebrates.

We had previously hypothesized that PRDM9 plays a role in directing recombination not only in mammals but across vertebrates, based on the presence of an intact ortholog across vertebrates with a rapidly evolving ZF (11). Consistent with our prediction, there is tentative evidence for the influence of PRDM9 binding on recombination in rattlesnakes (24). That a gene with a known role in recombination, *ZCWPW1*, coevolves with *PRDM9* across vertebrates lends further support to this hypothesis.

The precise nature of the molecular interactions between PRDM9 and ZCWPW1 remains unknown, but recent evidence suggests that ZCWPW1 interacts with PRDM9 to facilitate the repair of PRDM9-dependent DSBs: notably, *Zcwpw1*^−/−^ male mice and older female mice are sterile (27, 40) and exhibit defects in their ability to repair DSBs (25–27). In turn, the genomic locations of DSBs are not altered in *Zcwpw1*^−/−^ mice, indicating that the gene does not play a role in DSB positioning (25–27). In light of these experimental results, the coevolution of *PRDM9* with *ZCWPW1* across vertebrates indicates that PRDM9 likely plays a role in the efficient repair of DSBs not only in mice and humans (25–27, 41) but across the vertebrate phylogeny.

### Interpreting the Coevolution of *ZCWPW1* and *ZCWPW*2 With *PRDM9*.

If a gene interacts with PRDM9 by reading its histone modifications, as is the case for ZCWPW1 (25–27) and likely ZCWPW2 (Fig. 4), and has no other roles, we would expect that gene to be dispensable in species that no longer have an active PRDM9 SET domain. Previous papers reported that *ZCWPW1* is more likely to be missing from ray-finned fish with substitutions in catalytic tyrosine residues of the SET domain, in addition to clades lacking the entire *PRDM9* gene (26, 27). In our analysis, we find that both *ZCWPW1* and *ZCWPW2* are more likely to be absent from species carrying only *PRDM9* orthologs with substitutions in at least one catalytic tyrosine residue, as well as those lacking *PRDM9* altogether (Fig. 3).

While this pattern suggests a dependence of *ZCWPW1* and *ZCWPW2* on the intact catalytic activity of PRDM9, the interpretation is complicated by the fact that all species with substitutions at the tyrosine residues in all *PRDM9* copies are also carrying only partial *PRDM9* orthologs lacking KRAB and SSXRD domains, and nearly all species with conserved tyrosine residues also carry a complete copy of *PRDM9*. In that regard, the few exceptions are informative: Among species with confident *PRDM9* calls, the platypus and siluriformes fish carry *PRDM9* orthologs putatively missing the KRAB domain but with intact tyrosine residues. *ZCWPW2* is absent from all three siluriformes fish species analyzed here, while *ZCWPW1* is absent from one. Moreover, our results suggest a reduction of selective constraint on both *ZCWPW1* and *ZCWPW2* in platypus. Thus, the presence of *ZCWPW1* and *ZCWPW2* may depend on that of the KRAB domain rather than, or in addition to, the tyrosine residues remaining intact.

We note that although ZCWPW1 and ZCWPW2 are absent in the majority of lineages without complete PRDM9 genes, they are sometimes retained after the loss of PRDM9. Given the strong statistical evidence for coevolution, this retention could reflect pleiotropic constraints, if the genes play additional roles beyond their interaction with PRDM9, or simply that the genes have not yet been lost despite no longer being under constraint. In that regard, finding ZCWPW1 present and under selective constraint in siluriformes fish, *Xenopus* frogs, the green anole, and canids suggests that ZCWPW1 is not always dispensable in the absence of a complete PRDM9 gene. For instance, in a subset of species, *ZCWPW1* might retain a role in DSB processing or repair regardless of whether DSBs are initiated at PRDM9-bound sites.

In turn, the only species for which ZCWPW2 is retained in the clear absence of a complete PRDM9 are platypus and canids. These two cases are the most recent losses of PRDM9 in our analysis, and there is a relaxation of constraint on ZCWPW2 in both lineages, with ZCWPW2 appearing to evolve neutrally in canids. These findings suggest that ZCWPW2 is dispensable in the absence of a complete PRDM9 ortholog.

The molecular function of ZCWPW2 is to our knowledge unknown. Like its paralog, it could be involved in the processing or repair of DSBs. If so, the observation that *Zcwpw1*^−/−^ mice show defective DSB processing and repair (25–27) suggests that the role of ZCWPW2 cannot be completely redundant with that of its paralog. Alternatively, by reading the dual marks laid down by PRDM9, ZCWPW2 might help to recruit the recombination machinery (in particular SPO11) and thus play an earlier role in the positioning of DSBs. While in yeast, the link between histone modifications (specifically, H3K4me3) and the recruitment of Spo11 is made by Spp1 (42), in mammals, the ortholog of Spp1, CXXC1, is not essential for meiosis (43), and the gene that plays the analogous role has not yet been identified. Our analysis highlights ZCWPW2 as a potential candidate for this role, to be tested experimentally.

If ZCWPW2 does help recruit the recombination machinery, then losses of *ZCWPW2* could drive changes in recombination strategy across vertebrates. Indeed, we previously hypothesized that changes in recombination strategy from PRDM9-directed recombination to recombination occurring preferentially around promoter-like features arise by PRDM9 loss of function (11). If the key molecular interactors of PRDM9 have no pleiotropic rules, as may be the case for ZCWPW2 and in some lineages at least ZCWPW1, then it is also possible that their loss could also result in a switch in the way recombination is directed to the genome.

### Possible Links With TEX15 and FBX047.

The evidence for the coevolution of *TEX15* and *FBXO47* with *PRDM9* is much weaker, and neither appears dispensable in the absence of a complete *PRDM9* ortholog. If these two genes are indeed coevolving with *PRDM9*, the relationship is likely to be indirect. As a possible example, recent work implicates TEX15 as an effector of piRNA-mediated transposable element (TE) methylation and silencing (44, 45). Male mouse knockouts of *Tex15* exhibit a meiotic arrest phenotype associated with the failure to repair DSBs and to undergo chromosomal synapsis (41), as well as the transcriptional activation of TEs (44, 45), a phenotype similar to those observed in mouse knockouts of other piRNA-pathway genes, such as *Miwi* or *Dnmt3* (46). In *Dnmt3* knockout mice, it has further been shown that TEs accumulate both H3K4me3 marks and SPO11-dependent DSBs, suggesting that the methylation of TEs serves not only to silence them but may also result in preventing their use as sites of recombination (46). Thus, *TEX15* could conceivably play an indirect role in preventing the binding of PRDM9 to TEs.

In turn, *FBXO47* is a member of the F-box protein family, which act as recognition subunits of Skp1-Cullin1-F-Box protein E3 ubiquitin ligase complexes (38, 47). *FBXO47* has recently been implicated as a key regulator of the telomere shelterin complex during meiotic prophase I and in mice is necessary for telomere nuclear envelope attachment and subsequent events, including DSB repair (38). If this role of FBXO47 contributes to the formation of a chromatin environment that aids in the repair of PRDM9-dependent DSBs, or possibly in the recruitment of ZCWPW1, it might lead to increased conservation of *FBXO47* in the presence of *PRDM9*.

### Which Loss Came First?

While PRDM9 has two distinct roles—in specifying the location of DSBs and in facilitating their repair—the candidate genes that we have identified may only be involved in one of these two roles. If so, the dependencies between the presence of PRDM9 and of these genes could be asymmetric. For instance, if we ignore possible pleiotropic roles of ZCWPW1 and assume it is necessary for the repair of PRDM9 DSBs (but not DSB localization), we might predict that ZCWPW1 is likely to be lost after *PRDM9* (as appears to have been the case in *Tachysurus fulvidraco*; *SI Appendix*, Table S8*B*), as otherwise DSBs would go unrepaired. Whereas if ZCWPW2 is involved in DSB localization but not repair, it could be lost either before *PRDM9* (as was seemingly the case in two lineages of ray-finned fish; *SI Appendix*, Table S8*B*) or potentially after.

More generally, the phylogenetic data considered here do not allow us to distinguish between these scenarios: there is statistical evidence for a dependence of state transitions of *ZCWPW1*, *ZCWPW2*, *FBX047*, and *TEX15* on *PRDM9* as well as vice versa (in all tests, maximum *P* < 0.07, testing the null model of no dependence against either dependence as an alternative model; *SI Appendix*, Table S11). These scenarios could potentially be distinguished by collecting more fine-grained phylogenetic information to pinpoint the specific lineages in which the first loss occurred, as well as in light of further experimental data.

### Outlook.

Our phylogenetic analysis allowed us to identify putative interactors of PRDM9 that are promising candidates for functional studies. For this analysis, the power comes from the repeated losses of *PRDM9*—in our case, from 11 transitions from presence to absence. Confounding these kinds of analyses, however, are issues of data quality and in particular absences of complete *PRDM9* orthologs that reflect poor genome quality rather than true losses. To address this issue, we validated any absence in RefSeq with whole-genome searches and, when possible, de novo assemblies from RNA-seq data, leading us to realize that in one case (*MEI1*), the apparent coevolution with *PRDM9* was in fact spurious.

A more subtle but related issue stems from a phylogenetic signal of genome quality, which can lead to apparent clustering of losses. To minimize this issue, we restricted our analysis to genomes that included most “core” eukaryotic genes (*SI Appendix*, Fig. S7) and downsampled our tree to include at most three species below every inferred *PRDM9* loss. As genome qualities improve and as their assemblies become more uniform (e.g., Ref. 48), these issues should be alleviated. Moreover, as species are added to the phylogeny, additional losses will be identified; as one example, our identification of two species of frogs with a complete *PRDM9* revealed that *PRDM9* had not been lost once in the common ancestor, as had been inferred using fewer species by Baker et al. (11), but has instead been lost more than once within amphibians. This discovery also suggests that frogs may be an interesting clade within which to study the steps by which PRDM9 and its partners are lost.

Beyond the application to *PRDM9* and meiotic recombination, our analysis illustrates how long-standing phylogenetic approaches can now be applied to comparative genomic data to identify novel molecular interactions (49). Such analyses need not be restricted to measurements of presence or absence of whole genes, as we have done here, but could focus exclusively on specific domains, indicative of specific subfunctions, or consider how rates of evolution in specific domains depend on the presence or absence of other genes. With the explosion of high quality and more representative sets of genomes now coming online (e.g., Refs. 48–50), and the development of statistical methods that consider both binary and continuous character evolution jointly, we expect this type of approach to become increasingly widespread.

## Methods

### Identification of PRDM9 Orthologs.

We characterized the distribution of *PRDM9* in vertebrates following the same general approach as in our previous analysis (11, 12); a full description is provided in the *SI Appendix*. In brief, we first identified putative PRDM9 orthologs using a *blastp* search (30) against the RefSeq database and confirmed the orthology of each by visually inspecting where these genes clustered in neighbor-joining trees built with Clustal Omega (51) for identified KRAB, SSXRD, and SET domain sequences (*SI Appendix*, Fig. S1). For each species for which we could not initially identify a PRDM9 ortholog with KRAB and SET domains from RefSeq, we sought to identify PRDM9 orthologs from the nonredundant protein sequence database, whole-genome sequences, or testis RNA-seq datasets when available. We additionally searched whole-genome sequences or testis RNA-seq datasets from select species not represented in RefSeq in order to better time putative loss events. Lastly, we added to this dataset a set of PRDM9 orthologs previously identified from species not examined directly here (11, 12).

Altogether, this pipeline resulted in the identification of 202 species in which we find a complete *PRDM9* ortholog containing KRAB, SSXRD, and SET domains; 19 species for which we identify *PRDM9* orthologs containing KRAB and SET domains, but not SSXRD domains; 215 species for which we have evidence for the absence of a complete *PRDM9* gene; and 10 species for which we were unable to make a confident determination (Fig. 1; *SI Appendix*, Tables S1–S4). For each of the *PRDM9* orthologs that we identified, we characterized the conservation of three key tyrosine residues that have been shown to underlie the catalytic function of the SET domain (10, 52) and examined the evidence for positive selection acting on the DNA-binding specificity of PRDM9 ZF arrays following our previous approach (11, 12); see *SI Appendix* for details.

### Verification of Genomic Calls Using RNA-seq Data.

For four species in which we identified no *PRDM9* ortholog or only a partial ortholog, we investigated whether a complete *PRDM9* ortholog may nonetheless be present using RNA-seq data. We therefore sought to verify its absence from *A. carolinensis*, a species in which we had been unable to find a *PRDM9* ortholog in the genome assembly or RefSeq, as well as a second reptile species, *S. undulatus*, for which RefSeq data and a genome sequence were not available. To this end, we built a de novo RNA transcriptome assembly and tested for the expression of PRDM9 in testis and other tissue samples. Detailed information about these analyses can be found in *SI Appendix*.

Similarly, in two species in which we had originally identified only a partial ortholog of *PRDM9* (*Astyanax mexicanus* and *C. harengus*), we wanted to verify the incomplete domain structure inferred from the genome sequence by conducting a de novo transcriptome assembly (in *C. harengus*, this analysis turned out to be unnecessary, as an updated reference genome, GCA_000966335.1, contains a complete *PRDM9*). To this end, we analyzed RNA-seq data from *A. mexicanus* testis tissue and liver and testis from *C. harengus*. See *SI Appendix* for more details.

Using the same approach to de novo assembly and gene detection, we also analyzed publicly available RNA-seq datasets from testis for 28 additional species (*SI Appendix*, Table S3), either to verify the absence of PRDM9 or of one of the candidate genes; see *SI Appendix* for details.

### Choice of Candidate Genes and Orthology Assignments.

To identify a set of genes that may coevolve with *PRDM9*, we relied on three publicly available datasets, namely 1) 39 genes associated with variation in recombination phenotypes in a genome-wide association study in humans (33). Of the variants reported to be associated with recombination phenotypes, six were found in intergenic regions; we included the subset of two cases in which the authors assigned these variants to nearby genes (*ZNF84* and *ZNF140*); 2) 193 genes coexpressed with PRDM9 in single-cell data from mouse testes. Specifically, we considered the top 1% of genes based on their gene expression loadings in component 5, the component in which PRDM9 has the highest loading (31); and 3) 36 genes known to have a role in mammalian meiotic recombination based on functional studies (32).

Genes coexpressed with PRDM9 in mouse spermatogenesis were converted to human gene symbols using the package biomaRt in R (53). Fifteen of these genes did not have an orthologous human gene symbol (*Gm7972*, *H2-K1*, *Gm4349*, *Ddx43*, *Atad2*, *Xlr4c*, *Gm364*, *Tex16*, *4933427D06Rik*, *AI481877*, *H2-D1*, *Trap1a*, *Xlr4a*, *2310035C23Rik*, and *Tmem5*) and eight other genes mapped to more than one human gene symbol (*Msh5*, *Cbwd1*, *Nxf2*, *Cbwd1*, *Fam90a1b*, *Srgap2*, *Cdk11b*, *Gm15262*). Keeping all mapped gene symbols yielded 185 genes; combined with the two other sources, 241 genes were tested for their coevolution with *PRDM9* (*SI Appendix*, Fig. S6). A supplementary file describing each meiosis candidate gene is available in *SI Appendix*, Table S5.

For the 241 genes, we characterized whether the ortholog is present in its complete form across vertebrate species. To this end, we first downloaded all the vertebrate RefSeq protein sequences available on the NCBI database (accessed on June 3, 2020), corresponding to 339 species. Of these, we filtered out 32 species that were missing 10 or more BUSCO core genes (out of a total of 255 genes) (54), reasoning that their genomes were sufficiently incomplete that they may be missing orthologs by chance (*SI Appendix*, Fig. S7). Of the remaining 307 species, we further excluded 29 species in order to remove polytomies observed in the phylogeny; specifically, we removed the minimal number of species necessary to remove each polytomy while preserving any transitions in the state of *PRDM9*. Moreover, to minimize possible phylogenetic signals generated by genome assembly quality, we thinned the tree such that for each *PRDM9* loss along the phylogeny, we kept at most three species representing that loss. In cases in which a loss was ancestral to more than three species in our dataset, we picked three distantly related species with the best genome assemblies, as measured by the BUSCO score. In the end, we retained 189 species: 134 mammals, 3 birds, 6 amphibians, 18 reptiles, 2 percomorph fish, 3 cypriniformes fish, 20 other ray-finned fish, 2 cartilaginous fish, and 1 jawless fish. This phylogeny includes representative species for 11 of the 13 inferred PRDM9 losses; species of *Bufonidae* frogs and salamanders were not included because of the absence of available gene annotations; also because of the lack of gene annotations for frog species with PRDM9, within these 189 species, the losses in *Xenopus* and *Dicroglossidae* frogs cannot be distinguished from a single event.

For each candidate gene in each species, we performed a blastp search of the human ortholog against the RefSeq database of the species and kept up to five top hits obtained at an e-value threshold of 1e−5. We inferred the domain structure of each hit using the Batch CD-Search (55) and considered a domain as present in a species if the e-value was ≤0.1. We considered genes to be complete orthologs if they contained the superfamily domains found in four representative species of the vertebrate phylogeny carrying a complete PRDM9 [*Homo sapiens* (human), *Esox lucius* (fish), *Geotrypetes seraphini* (caecilian), and *Pseudonaja textilis* (snake)], at an e-value threshold of 1e−4. For the 15 genes (*FANCB*, *FMR1NB*, *GPR137C*, *HAUS8*, *M1AP*, *MEI1*, *SPATA22*, *CLSPN*, *FBXO47*, *HMGA2*, *HSF2BP*, *IQCB1*, *LRRC42*, *PRAME*, *SYCE2*) in which no detectable domains were present, we annotated the presence or absence of the gene using the blastp results alone. In the end, we built a matrix of presence or absence across species and candidate genes to be used in the phylogenetic test (*SI Appendix*, Table S6).

### Testing for the Coevolution of PRDM9 and Candidate Genes.

To test for the coevolution of *PRDM9* and each candidate gene, we need to account for the phylogenetic relationships among the species considered. To obtain these relationships and time-calibrated branch lengths, we used the TimeTree resource (http://timetree.org/; Ref. 35; accessed on June 10, 2020). Of the 189 species included in the phylogenetic tests, nine were not present in the TimeTree database; in those cases, we used information from a close evolutionary relative to determine their placement and branch lengths.

For this test, we consider *PRDM9* as present if it contains the KRAB and SET domains or incomplete/missing if one of those domains is absent (*SI Appendix*, Tables S4 and S8). We do not rely on the SSXRD domain when making these calls because its short length makes its detection at a given e-value threshold unreliable. Notably, for 19 of the 26 species with *PRDM9* orthologs containing KRAB and SET domains, but not SSXRD domains, with an e-value < 1, we are able to detect the SSXRD domain when using an e-value threshold of 1,000 (*SI Appendix*, Table S1). We additionally do not rely on the ZF array because its repetitive nature makes it difficult to sequence reliably.

We tested whether state changes of intact candidate genes were unexpectedly coincident with state changes of the intact *PRDM9* using the software *BayesTraitsV3* (56). We did so by comparing the statistical support for two models: a null model in which *PRDM9* and a given candidate gene evolve independently of one another along the phylogeny versus an alternative model in which the gain (“1”) and loss (“0”) of a gene is dependent on the status of *PRDM9* and vice versa. We compared the likelihoods of the two models using a likelihood ratio test with four degrees of freedom and reported a *P* value uncorrected for multiple tests (*SI Appendix*, Table S7). For each gene and model, 100 maximum likelihood tries were computed, and the maximum likelihood value was retained. A quantile-quantile plot was drawn to access the distribution of *P* values, and the R package “Haplin” was used to compute pointwise confidence intervals. To control for the FDR, we computed q-values using the R package “qvalue” and set a 50% FDR threshold.

Given the phylogenetic distribution of *PRDM9*, it is likely that a *PRDM9* ortholog was present in the common ancestor of vertebrates (11, 12). Based on this prior knowledge, we restricted the state of *PRDM9* at the root of the phylogeny to always be present. In turn, for each candidate gene, we set a prior in which it had 50% probability of being present and 50% probability of being absent. We also used this prior for the state of *PRDM9* in the nine species that lack *PRDM9* but in which the loss was not supported by a closely related species (i.e., for which we considered the status uncertain).

For *FBXO47*, *TEX15*, *ZCWPW1*, and *ZCWPW2*, we also explored restrictions on the rates in the dependent model such that their state transitions depend on PRDM9 (model X) or the state transitions of *PRDM9* depends on theirs (model Y), rather than both being true. For these tests, we compared the likelihoods of each dependent model against our independent null model using a likelihood ratio test with two degrees of freedom. For each gene and model, 100 maximum likelihood tries were computed, and the maximum likelihood value was retained.

We also explored whether redefining a complete PRDM9 ortholog as containing not only the KRAB and SET domain but also the SSXRD domain would change the statistical significance. By using the improved calls (see below), only *ZCWPW2* remains significant (*P* = 0.004) and *ZCWPW1* marginally so (*P* = 0.056) (*SI Appendix*, Table S10 *B* and *C*).

In addition, we considered the coevolution of PRDM9 with genes highly expressed in the component in which PRDM9 had its second highest loading in single-cell data from mouse testes (31). For this set, the *P* values were roughly uniform, as expected under the null model of no coevolution, and no gene stood out as a promising candidate (*SI Appendix*, Fig. S12).

### Improving Gene Status Calls of Top Candidate Genes.

For the five genes with an FDR ≤ 50% (Fig. 2*A*), we sought to improve our calls by building phylogenetic trees based on domains in the genes and examining the clustering patterns visually, as well as by searching for orthologs in whole-genome assemblies and testis transcriptomes (following the same procedures described for *PRDM9*). These improved calls were then used to rerun the phylogenetic independent contrast tests, following the same implementation as previously; the *P* values for these improved gene models are shown alongside the original ones in Table 1. We provide an overview of the steps taken for each candidate gene in the *SI Appendix*. For each gene, we provide descriptions of identified orthologs and how they were identified in *SI Appendix*, Table S1; specific details about orthologs identified from whole-genome assemblies in *SI Appendix*, Table S2; our improved calls per species in *SI Appendix*, Table S8*A*; and a summary of loss events in *SI Appendix*, Table S8*B*.

### Conservation of Residues in *ZCWPW2*.

We carried out a residue conservation analysis using an approach proposed by Ref. 57, using code *score_conservation.py* available at https://compbio.cs.princeton.edu/conservation/. This approach quantifies the Jensen-Shannon divergence between the amino acid distribution of the focal residue and a “background amino acid distribution.” The alignment of *ZCWPW2* was produced using Clustal Omega (using default parameters) within MEGA (version 7) (35, 58). As recommended, the overall background amino acid distribution was drawn based on the BLOSUM62 amino acid substitution matrix provided by the software (57). Any column of the gene sequence alignment with more than 30% gaps was ignored. A window size of 3 was used to incorporate information from sequential amino acids, as recommended by the default settings.

### Evidence for Relaxed Selective Constraint in the Absence of *PRDM9.*

To test for possible relaxed selection in species without a complete *PRDM9*, we used the program *codeml* within PAML (59, 60). *Codeml* uses protein coding sequences to estimate the ratio of nonsynonymous to synonymous substitution rates (ω = *d_N_*/*d_S_*). Values of ω significantly less than 1 are indicative of purifying selection, i.e., of the functional importance of the gene.

To this end, we considered each major clade (fish, mammals, reptiles, amphibians) separately and extracted and aligned coding nucleotide sequences from NCBI for multiple species. We aligned those sequences in a codon-aware manner using Clustal Omega (using default parameters) within MEGA (version 7; Refs. 35, 58) and inspected the codon-aware alignment visually to ensure that the same isoforms were used across species. For each multispecies alignment, we tried two approaches. 1) We estimated ω under a null model assuming the same ω across all branches and an alternative model in which there are two ω allowed: one ω value in species with a complete *PRDM9* and a second ω for the branches in which *PRDM9* is absent or incomplete (including the internal branches on which *PRDM9* may have been lost). 2) We considered the same null model with the same ω across all branches and an alternative model with one ω value in species with a complete *PRDM9*, a second ω for the branches in which *PRDM9* is absent or incomplete, and additional ω values for each branch on which *PRDM9* was inferred to be lost (a different one for each independent loss, as the ω value averaged over the branch will depend on when along the branch *PRDM9* was lost). For 1), significance was assessed using a likelihood ratio test with one degree of freedom; for 2), by the number of degrees of freedom corresponded to the number of distinct ω values minus one. If ω values were found to be significantly higher in species without a complete *PRDM9*, we tested whether or not we could reject ω = 1 for these species. For two cases in which we could not obtain a multispecies alignment that included the whole coding sequence (*ZCWPW1* in fish and *TEX15* in amphibians), we instead used the pairwise model (runmode: -2 within PAML) on alignments for a pair of species and tested whether we could reject ω = 1 for species lacking *PRDM9* by comparing a model allowing ω to vary versus a null model fixing the ω value at 1, with one degree of freedom.

## Supplementary Material

Supplementary File

## Data Availability

RNA-seq data have been deposited in NCBI, ID: PRJNA605699. Code generated for this study can be found in GitHub at https://github.com/izabelcavassim/PRDM9_analyses. All other study data are included in the article and/or supporting information.
