# Supplementary material for: PRDM9 losses in vertebrates are coupled to those of paralogs ZCWPW1 and ZCWPW2
Source: Proc Natl Acad Sci U S A. 2022 Feb 25;119(9):e2114401119. doi: 10.1073/pnas.2114401119 (PMC8892340; doi:10.1073/pnas.2114401119)
Supplement: Supplementary File [file pnas.2114401119.sapp.pdf]

Supplementary tables

Supplementary Table 1. Genes identified in this study. For *PRDM9* and each candidate gene that was initially identified as significantly coevolving with *PRDM9* (*ZCWPW1*, *MEI1*, *ZCWPW2*, *TEX15*, and *FBX047*), we provide a table detailing, for each ortholog that we identified, which species it is from, how we identified it, its inferred domain architecture, its amino acid sequence, as well as various details about these domains (including their coordinates, e-values, and sequences). For each *PRDM9* ortholog, we additionally report which amino acid residues align to three catalytic tyrosine residues in the human SET domain, as well as the proportion of amino acid diversity observed at DNA-binding residues in an alignment of ZFs, how many ZFs were used in these alignments, and the ranking of this statistic across other C2H2-ZF genes from the same species.

Supplementary Table 2. Description of genes found from whole genome sequences. For *PRDM9* and each gene initially identified as significantly coevolving with *PRDM9* (*ZCWPW1*, *MEI1, ZCWPW2*, and *TEX15*), we provide a table detailing where and how each ortholog identified in our analysis of whole genome assemblies was obtained. *FBXO47* is excluded from this table because no *FBXO47* orthologs were identified from whole genome sequences.

Supplementary Table 3. Description of species for which a *de novo* assembly of testis transcriptomes was generated in order to verify the structure and expression of PRDM9 and four significant genes (ZCWPW1, ZCWPW2, TEX15 and FBX047). To this end, we used publicly available RNA-seq data (downloaded from NCBI) (1) and in a subset of cases indicated with a star, generated our own data which are available from the NCBI sequence read archive (Bioproject PRJNA605699, SRA accessions: SRR11050679-SRR11050687, see Methods for further details).

Supplementary Table 4. The distribution of *PRDM9* orthologs across 446 vertebrate species. For each species, we describe how many *PRDM9* orthologs we identified of each unique domain architecture, the domain architecture of the most complete *PRDM9* ortholog from that species, whether any *PRDM9* ortholog from that species with the most complete domain architecture has conserved three catalytic tyrosine residues in the SET domain, whether any PRDM9 ortholog from that species shows evidence of rapid evolution of its ZF array, as well as the accessions of each non-PRDM9 C2H2-ZF gene used to generate species-specific empirical distributions of our statistic for rapid evolution. We additionally include columns comparing these results to those previously described in Baker et al. 2017, noting instances where we have revised our calls of domain architecture.

Supplementary Table 5. Description of candidate genes used in the phylogenetic tests. The 241 genes selected for the tests were based on three different sources: (source 1) genes most highly co-expressed with PRDM9 in mouse testis single cell analyses (2), (source 2) genes associated with variation in recombination phenotypes in humans (3) (i.e.,“crossover locations” and “recombination rate”), and (source 3) genes known to have a role in mammalian meiotic

recombination from functional studies (as summarized in the review by (4)). The genomic coordinates (column named "Start position") of each gene were based on the GRCh38/hg38 human reference; for source 3, we provide the Start position of the nearest gene. The Category of each candidate gene is described based on the definition from its source.

Supplementary Table 6. Presence and absence matrix computed for all candidate genes used in phylogenetic tests for coevolution with *PRDM9* (139 genes). We defined a gene as complete ("1") when it contained all the domains observed in four representative vertebrate species with a complete *PRDM9* sequence, and incomplete ("0") if the gene was not detected in the Refseq database or if it did not include all the domains shared across four species (see Methods for details).

Supplementary Table 7. Phylogenetic tests and p-values. P-values were computed by evaluating the patterns of presence or absence of *PRDM9* across 189 vertebrates against the patterns of presence or absence of candidate genes. Two models were tested using BayestraitsV3 (5): a null model in which *PRDM9* and a given candidate gene evolve independently of one another along the phylogeny versus an alternative model in which the gain ("1") and loss ("0") of the candidate gene is dependent on the status of *PRDM9* and vice versa. See Pagel, 1994 and the BayesTraitsV3.0.2 manual for further discussion of these models and rates description.

Supplementary Table 8. The distribution of PRDM9 and four genes initially found to be significantly coevolving with *PRDM9* (*ZCWPW1*, *ZCWPW2, TEX15*, and *FBXO47*) across 189 vertebrate species**.** *MEI1* is not considered because in the curation of the calls, it was found to be present in all species (see text). (**A**) Curated calls for the presence or absence of complete genes based on searches of RefSeq, whole genome assemblies, and RNA-seq data (see **Tables S1-S3**). We additionally include the most complete domain architecture of orthologs from each species for each gene. (**B**) Summary of losses inferred for *PRDM9*, *ZCWPW1*, *ZCWPW2*, *TEX15 and FBXO47* among the 189 vertebrate species used in our co-evolutionary test.

Supplementary Table 9. Tests for differences in the rates of amino acid evolution in three significant genes (*ZCWPW1*, *ZCWPW2*, *TEX15* and *FBX047*) between representative species with and without a PRDM9 ortholog. To determine whether species lacking a *PRDM9* ortholog showed evidence for relaxed selection pressures in co-evolving genes, we estimated ω(dN/dS) using the Branch model within PAML (6) under two models: a null model assuming the same ω across all branches of the phylogeny, and an alternative model in which there are two ω values allowed: one ω value in species lacking a functional PRDM9 and a second ω for the rest of the branches. The clades evaluated in each test are specified. The species used in the alignment for each test are also shown. The log likelihoods for each model, ω estimates and p-values are also provided. See Methods for details.

Supplementary Table 10. (**A**) Results of phylogenetic tests when considering the pairwise co-evolution of the candidate genes with each other. (**B**) Results of phylogenetic tests when considering the SSXRD domain in *PRDM9* classification. P-values were computed by

evaluating the patterns of presence or absence of *PRDM9* across 189 vertebrates against the patterns of presence or absence of candidate genes. Two models were tested using BayestraitsV3 (5): a null model in which *PRDM9* and a given candidate gene evolve independently of one another along the phylogeny versus an alternative model in which the gain ("1") and loss ("0") of a gene is dependent on the status of *PRDM9* and vice versa. See (7) and the BayesTraitsV3.0.2 manual for further discussion of these models and rates description. (**C**) Results of phylogenetic tests when considering the SSXRD domain in *PRDM9* classification and the curated calls for *ZCWPW1*, *ZCWPW2, TEX15*, and *FBXO47.*

Supplementary Table 11. Testing the direction of dependency between *PRDM9* and candidate genes *ZCWPW1*, *ZCWPW2, TEX15* and *FBX047*. Here, we asked whether we could reject a model of independent state transitions of *PRDM9* and a given candidate gene (e.g. *ZCWPW1*) in favor of a model in which state transitions of the candidate gene depend on those of *PRDM9* (model X). Next, we asked whether we could reject the null model in favor of a model in which the state transitions of *PRDM9* depend on those of the candidate gene (model Y). For comparison, we also provide results for the test shown in the main text, in which the alternative considered is that state transitions of *PRDM9* depend on those of the candidate gene and vice versa (also shown in Table 1). See Pagel, 1994 and the BayesTraitsV3.0.2 Manual for further description of these models and tests.

Supplementary Information

1. Identification of PRDM9 orthologs

As a first step towards characterizing the distribution of *PRDM9* in vertebrates, we identified putative *PRDM9* orthologs in the RefSeq database with a *blastp* search (30), using the N-terminal portion of the *Homo sapiens* PRDM9 protein sequence containing KRAB, SSXRD and SET domains as the query sequence (RefSeq accession: NP_001297143; amino acid residues 1-364). We downloaded the corresponding GenBank file for 5,000 hits (3,400 unique genes from 412 species) and characterized the presence or absence of KRAB, SSXRD and SET domains for each record using the Conserved Domain, Protein Families, NCBI curated and SMART databases (CDD (8); Pfam (REF); NCBI curated (REF); SMART (REF); accessions cl02581 and cl09744 for the KRAB and SSXRD domains respectively, and accessions cl40432 and cl02566 for the SET domain), annotating each domain as present if that domain had an e-value less than 1 in any of the four databases. We then removed alternative transcripts from the dataset by preferentially keeping, for each unique gene, the transcript with the maximal number of annotated domains. When there were multiple transcripts with the same maximal number of domains, we kept the longest one.

Because *PRDM9* shares its SET domain with other PRDM family genes and its N-terminal domains with members of the KRAB-ZF and SSX gene families, many of these hits

are potential PRDM9 paralogs. To identify bona fide *PRDM9* orthologs from this initial set of genes, we sought to build phylogenetic trees specific to the KRAB, SSXRD, and SET domains and remove homologs that cluster with genes annotated as distantly related paralogs of *PRDM9*. To this end, we extracted the amino acid sequences for complete KRAB, SSXRD, and SET domains, and for each domain, constructed neighbor-joining trees using Clustal Omega (9). Utilizing the KRAB and SSXRD domain-based trees, we identified and removed 87 genes that visually cluster with members of the SSX gene family (**Figure S1A-B**). Analyzing the SET domain-based tree, we identified and removed 2,637 genes that group with other members of the *PRDM* gene family (**Figure S1C**; see figure legend for details). We ultimately retained 625 genes, each of which cluster with *PRDM9* in one or more of these trees.

By this approach, in the 412 species considered, we identified 209 *PRDM9* orthologs containing KRAB, SSXRD and SET domains from 155 species, as well as 13 *PRDM9* orthologs containing KRAB and SET domains for which we were unable to detect an SSXRD domain with an e-value less than 1 from an additional 11 species. For the 246 species for which we were unable to identify a *PRDM9* ortholog spanning KRAB and SET domains in our initial search of the RefSeq database, we sought to verify that *PRDM9* was truly absent using a number of approaches.

As a first step, we performed an additional blastp search against the non-redundant protein sequence (nr) database, targeting only those species in order to identify any annotated gene record missed in our initial search of the RefSeq database. We downloaded the corresponding GenBank file for each hit with >55% coverage and >40% identity and, after removing records corresponding to those we had previously identified, annotated domains and removed alternative transcripts as before. We then verified the orthology of the remaining records by blasting each protein sequence against the human RefSeq database, accepting it as a PRDM9 ortholog if the top hit was PRDM9 or its paralog PRDM7. This approach enabled the identification of an additional 9 PRDM9 orthologs, including one containing KRAB, SSXRD and SET domains, and one containing KRAB and SET domains.

Next, we performed a series of *tblastn* searches of the whole genome of the 244 species remaining using the N-terminal portion of the *Homo sapiens* PRDM9 protein as a query. When we were unable to retrieve any promising hits with the human protein sequence, we re-performed the *tblastn* search using the N-terminal portion of a *PRDM9* ortholog from a species closely related to the focal species. In order to identify which of the identified contigs corresponded to genuine *PRDM9* orthologs (as opposed to paralogs such as *PRDM11*), we performed blastp searches against the *Homo sapiens* RefSeq database using the aligned

protein sequences as query sequences. Contigs containing the relevant alignments spanning KRAB and/or SET domains were then downloaded and the aligned region including 10,000 of flanking sequence was extracted and input into *Genewise (10)*, using the PRDM9 protein sequence from *Homo sapiens* or a closely related species as a guide sequence (see **Table S2** for details). In genomes from 10 species, we identified separate contigs containing the KRAB domain and the SET domain. In these cases, the contigs were concatenated before use as input in *Genewise*. These approaches enabled us to identify an additional 53 *PRDM9* orthologs from 33 species, including 21 *PRDM9* orthologs containing KRAB, SSXRD and SET domains from 21 species, and 24 *PRDM9* orthologs containing KRAB and SET domains but for which we were unable to identify the SSXRD domain from 11 species.

These analyses left 210 species for which we were unable to identify a *PRDM9* ortholog with both KRAB and SET domains. For these species, with the exception of 94 birds and crocodiles and 78 percomorpha fish, where the absence or truncation of *PRDM9* has been previously demonstrated, we additionally searched testis RNA-seq datasets when possible, including those generated for this study (see below; **Table S3**). This approach enabled us to identify two additional *PRDM9* orthologs containing KRAB and SET domains from two species of fish.

From this analysis, and given the phylogenetic relationships among species given by the TimeTree tool (11), we inferred 20 putative complete or partial losses of PRDM9 across the 412 species represented in the RefSeq database. Of these, 7 losses were supported by the absence of PRDM9 in two or more closely related species: in percomorpha and beryciformes fish, characiformes and siluriformes fish, cypriniformes fish, polypteridae fish, frogs, birds and crocodiles, and canids.

The remaining 13 inferred losses each corresponded to an individual species. In order to identify whether or not any of these 13 latter absences could be supported by additional species, and to more accurately infer the dates of each loss, we sought to investigate the status of PRDM9 in species closely related to each putative loss event. To this end, we investigated the whole genomes of an additional 18 species and RNA-seq datasets from an additional 4 species as before, with one species represented by both a whole genome sequence and a corresponding RNA-seq dataset (*Ambystoma mexicanum*). This approach enabled us to identify an additional 6 PRDM9 orthologs containing KRAB, SSXRD and SET domains from 6 species, as well 15 additional species putatively lacking a complete PRDM9 gene. In doing so, we found that 2 additional losses were supported by the absence of PRDM9 in two or more closely related species: in osteoglossomorpha fish, as well as a loss within lizards shared by *Anolis*

carolinensis and *Sceloporus undulatus*. Moreover, we identified two species of frogs carrying complete PRDM9 orthologs. This discovery suggests that PRDM9 has been lost repeatedly within amphibians – at least once in salamanders, and at least three times within frogs (with each of these four putative loss events being supported by the absence of the PRDM9 in two or more closely related species).

For each of the 11 remaining instances in which only a single species was found to be lacking PRDM9, the most closely related species considered possessed a complete PRDM9 ortholog. While we were able to confirm the absence of a complete PRDM9 in platypus using RNA-seq data (see below), we do not have confirmatory evidence of absence for the remaining 10 species, and therefore treat these species as having an uncertain PRDM9 status.

Lastly, we include in the list of species considered an additional 13 species for which we had previously identified complete PRDM9 orthologs (12) but which were not directly examined here (**Table S4**). Altogether, this pipeline resulted in the identification of 202 species in which we find a complete *PRDM9* ortholog containing KRAB, SSXRD and SET domains, 19 species for which we identify *PRDM9* orthologs containing KRAB and SET domains but not SSXRD domains, 215 species for which we have evidence for the absence of a complete *PRDM9* gene, and 10 species for which we were unable to make a confident determination (see **Tables S1-S4, Figure 1**).

For each of the *PRDM9* orthologs that we identified, we characterized the conservation of three key tyrosine residues that have been shown to underlie the catalytic function of the human SET domain in vitro (i.e., Y276, Y341, and Y357; (13)) and for Y357, in vivo in mouse (14). To this end, we constructed an alignment of the SET domain using Clustal Omega (9) and extracted the residues aligning to the human tyrosine residues from each of 678 SET domains (**Table S1**).

Lastly, we examined the evidence for positive selection acting on the DNA-binding specificity of PRDM9 ZF arrays. To this end, we calculated the proportion of amino acid diversity that is localized to the DNA binding residues within alignments of C2H2 ZFs found in each array, a statistic sensitive to both rapid turnover at DNA-binding residues and high rates of gene conversion between fingers ((12, 15–17)). In doing so, we considered only genes containing tandem arrays of four or more ZFs, requiring each to match the 28 amino acid long C2H2 motif exactly (X2-CXXC-X12-HXXXH-X5, where X is any amino acid). If a gene possessed multiple tandem arrays, only the first was considered. To assess the significance of our results, we compared the statistic generated for the PRDM9 ortholog to those of other C2H2 ZF genes from the same species (accessions provided in **Table S4**). Each PRDM9 ortholog was then ranked

against the non-PRDM9 C2H2 ZF genes identified from the same species, and assigned an empirical p-value based on this distribution (see **Table S1** and **Table S4**). Considering species for which we had at least 50 non-PRDM9 C2H2-ZF genes for comparison, we are unable to identify a rapidly evolving ZF array (at a nominal significance level of 0.05) in only three species carrying putatively complete PRDM9 orthologs (*Pteropus vampyrus*, *Chelonoidis abingdonii* and *Rhincodon typus*); conversely, we identified rapidly evolving ZF arrays from only three species for which we could not identify a complete PRDM9 ortholog (*Myotis lucifugus*, *Myotis davidii* and *Pangasianodon hypophthalmus*).

2. Verification of genomic calls using RNA-seq data

Dissected tissue samples preserved in RNAlater were kindly provided to us by Arild Folkvord and Leif Andersson (*Clupea harengus*), Cliff Tabin (*Astyanax mexicanus*), Tonia Schwartz and Tracy Langkilde (*Sceloporus undulatus*), and Athanasia Tzika (*Anolis carolinensis*). These samples were stored at -20°C until extraction and library preparation. Total RNA was extracted using the Qiagen RNeasy kit (Valencia, CA, USA) following the manufacturer's protocol. RNA was quantified and assessed for quality on a Qubit fluorometer and approximately 1 µg of total RNA was input for library preparation using the Kapa RNA-seq kit. Samples were prepared following the manufacturer's protocol, except that half reactions were used. Briefly, mRNA was purified using manufacturer's beads and chemically fragmented. First and second-strand cDNA was synthesized and end-repaired. Following A-tailing, each sample was individually barcoded with an Illumina index and amplified for 12 cycles. In order to evaluate the library quality and size distribution, libraries were evaluated on an Agilent Tapestation. The libraries were then sequenced over two runs on the NextSeq 550 at Columbia University to collect paired-end 150 bp reads.

Illumina sequencing reads (248,820,547 2x150 base pair (bp) paired-end reads) were demultiplexed into individual sample fastq files with the software bcl2fastq2 (v2.20.0, Illumina). The FastQC software (18) was used for visual inspection of read quality. Adapters and low-quality reads were trimmed with the Trimmomatic software, which is bundled as a plugin within the Trinity *de novo* assembler (19) (v2.8.5) and was enabled using the --*trimmomatic* flag. The default trimming settings (phredscore>=5; slidingwindow:4:5; leading:5, trailing:5; minlen:25) were used following (20) recommendations. The pair-end reads were trimmed and *de novo* transcriptomes assembled with Trinity (v2.8.5) using the following parameters: --seqType fq --SS_lib_type FR --max_memory 100G --min_kmer_cov 1 --trimmomatic --CPU 32. Details on assembly quality are shown in **Figure S2**. Gene expression data for all four species

(*Anolis carolinensis, Sceloporus undulatus, Clupea harengus, Sceloporus undulatus*) are available from the NCBI sequence read archive (Bioproject PRJNA605699, SRA accessions: SRR11050679-SRR11050687).

To evaluate whether PRDM9 was present in the transcriptome data, we conducted a *tblastn* search (e-value ≤ 1e-5) against each *de novo* assembly using the human PRDM9 protein sequence (without its rapidly evolving zinc finger array) as a query, and we classified the domain presence of up to five top hits using CDD blast (8). For a given species, if the KRAB and SET domains were not identified in any transcript, *PRDM9* was considered incomplete. The inability to identify PRDM9 could indicate either that the gene is not expressed or that we lack the appropriate cell types or sequence coverage to detect it. To assess our power to detect PRDM9 from the testis RNA-seq data, we followed methods outlined in (12). Specifically, for each transcriptome, we evaluated whether we could identify transcripts from six genes with highly conserved roles in meiotic recombination (21) (*HORMAD1*, *MEI4*, *MRE11A*, *RAD50*, *REC114*, and *SPO11*). To identify the transcripts orthologous to each of these genes, we performed a *tblastn* search (e-value ≤ 1e-5) of the *Homo sapiens* reference protein sequence against each *de novo* transcriptome. We considered PRDM9 to be absent if we detected expression of all six genes but not a complete *PRDM9*; by these criteria, we found PRDM9 to be missing from *A. carolinensis*, *S. undulatus*, and *A. mexicanus*.

To estimate the expression levels of the Trinity-reconstructed transcripts, we used RSEM (22) (v1.3.1) implemented through Trinity (v2.8.5). We first aligned the RNA-seq reads from each sample to the newly generated *de novo* assembled transcriptome (see above) using the alignment method bowtie (23) (v1.2.2). We then extracted quantification information for each gene of interest from the RSEM output (in fragments per kilobase of transcript per million mapped reads or FPKM) (**Figure S3**).

3. Improving gene status calls of top candidate genes

i. *MEI1*

For *MEI1*, an initial blastp search of the vertebrate RefSeq database using the human sequence as query resulted in the identification of 422 *MEI* orthologs from 372 species. We note that, for *MEI1*, we did not find any domain annotations, and therefore did not perform phylogenetic analysis to support the identification of these orthologs. However, each homolog identified in our initial RefSeq analysis was annotated as either *MEI1* or *MEI1-like*. We thus labeled each species as having a complete ortholog if an ortholog was present. This approach resulted in the identification of *MEI1* ortholog for 187 of the 189 species used for our

co-evolutionary test. For the remaining 2 species, we sought to identify *MEI1* orthologs from whole genome sequences following the same procedures described for *PRDM9*. This approach allowed us to identify a *MEI1* ortholog in every species, revealing that in fact, *MEI1* has not been lost among the vertebrate species examined (**Tables S1** and **S2**).

ii. *ZCWPW1* and *ZCWPW2*

Because *ZCWPW1* and *ZCWPW2* are paralogs, we performed our analyses of these genes together. To this end, we combined the datasets of genes identified in our initial RefSeq blastp search to create a dataset of 977 putative orthologs from 363 species. We then extracted amino acid sequences and built neighbor-joining trees using Clustal Omega for both the zf-CW and PWWP domains ((9); accessions cl06504 and cl02554; **Figure S9A-B**). Utilizing these trees, we removed 573 genes that visually clustered with genes annotated as distantly related paralogs, such as members of the MORC and NSD gene families. We additionally relied on these trees to more confidently label which genes were *ZCWPW1* orthologs and which were *ZCWPW2* orthologs based on where they clustered in the tree. We considered orthologs as complete if they contain both PWWP and zf-CW domains with e-values < 1. This approach resulted in the identification of 193 complete *ZCWPW1* orthologs from 188 species, and 187 complete *ZCWPW2* orthologs from 180 species.

Among the 189 species used in our co-evolutionary test, 164 had complete *ZCWPW1* orthologs and 154 had complete *ZCWPW2* orthologs on the basis of this initial search. For the 25 species missing a complete *ZCWPW1* ortholog, and for the 35 missing a complete *ZCWPW2* ortholog, we sought to identify the orthologs from whole genome sequences following the same procedures as described for *PRDM9*. This approach enabled us to identify an additional 3 complete *ZCWPW1* orthologs from 3 species, and an additional 11 complete *ZCWPW2* orthologs from 11 species (**Tables S1** and **S2**). For the remaining species, we checked the putative loss of *ZCWPW1* or *ZCWPW2* using RNA-seq data when available, which led to the identification of an additional 2 complete *ZCWPW1* orthologs, but no additional *ZCWPW2* orthologs (**Tables S1** and **S3**). We additionally added one ZCWPW1 ortholog from the common shrew (*Sorex araneus*) from the Ensemble database, which had been identified previously but was absent from NCBI (24, 25). Lastly, we sought to identify *ZCWPW2* from the whole genome sequence of a species of frog with PRDM9 (*Ranitomeya imitator*) not otherwise included in our co-evolutionary test in order to distinguish whether or not the absence of *ZCWPW2* in *Xenopus* and *Dicroglossidae* frogs corresponded to a single loss or multiple events. We were able to

identify *ZCWPW2* from this species, suggesting that *ZCWPW2* has been lost multiple times within frogs (**Tables S1**, **S2** and **S8**).

iii. *TEX15*

For *TEX15*, our initial blastp search resulted in the identification of 900 putative orthologs from 363 species. We similarly utilized a tree built using the DUF3715 domain to remove 667 genes that cluster with distantly related paralogs, in particular, *TASOR* and *TASOR2* (**Figure S9C**). When making our final calls about *TEX15* orthologs, we labeled them as complete if they contained both DUF3715 and TEX15 domains (accessions pfam12509 and pfam15326). This approach resulted in the identification of 179 complete *TEX15* orthologs from 175 species. Among the 189 species used for our co-evolutionary test, 150 had complete *TEX15* orthologs on the basis of this initial search. For the 39 species missing a complete *TEX15* ortholog, we sought to identify the orthologs from whole genome sequences, following the same procedures as described for *PRDM9*. In this way, we identified an additional 29 complete *TEX15* orthologs from 28 species (**Table S2**). For the remaining species, we checked if we could find *TEX15* using RNA-seq data, when available, and found one additional complete *TEX15* ortholog by this approach (**Tables S1** and **S3**).

iv. *FBXO47*

For *FBXO47*, an initial blastp search of the vertebrate RefSeq database using the human sequence as query resulted in the identification of 386 putative *FBXO47* orthologs from 380 species. We did not perform phylogenetic analysis to support the identification of these orthologs: While we detected a domain (F-BOX) in the human *FBXO47* gene, due to its high e-value in humans (e-value = 0.01), we did not rely on its presence or absence when inferring the whether or not a complete *FBXO47* gene was present in each species. However, each homolog identified in our initial RefSeq analysis was annotated as either *FBXO47* or *FBXO47*-like with the exception of one *CWC25* gene, which was removed. We thus labeled each species as having a complete ortholog if an ortholog was present. This approach resulted in the identification of *FBXO47* ortholog for 181 of the 189 species used for our co-evolutionary test. For the remaining 8 species, we sought to identify *FBXO47* orthologs from whole genome sequences and/or RNA-seq datasets following the same procedures described for *PRDM9*; however, we were unable to identify any additional FBXO47 in this way (**Tables S1** and **S3**).

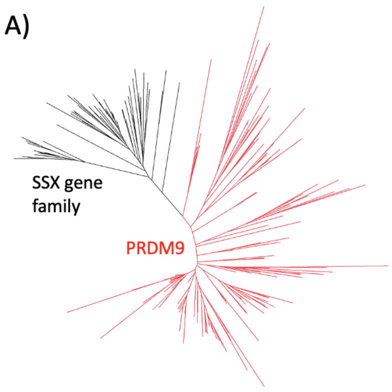

Figure S1. Guide trees created from our initial blastp search results for PRDM9 orthologs for **(A)** KRAB domains, **(B)** SSXRD domains and **(C)** SET domains. Genes were removed if they clustered with SSX genes in trees **(A)** or **(B)**, or if they clustered with PRDM gene family genes other than *PRDM9* or *PRDM7* in the tree **(C)**. Genes clustering with *PRDM9* and retained for subsequent analysis are shown in red.

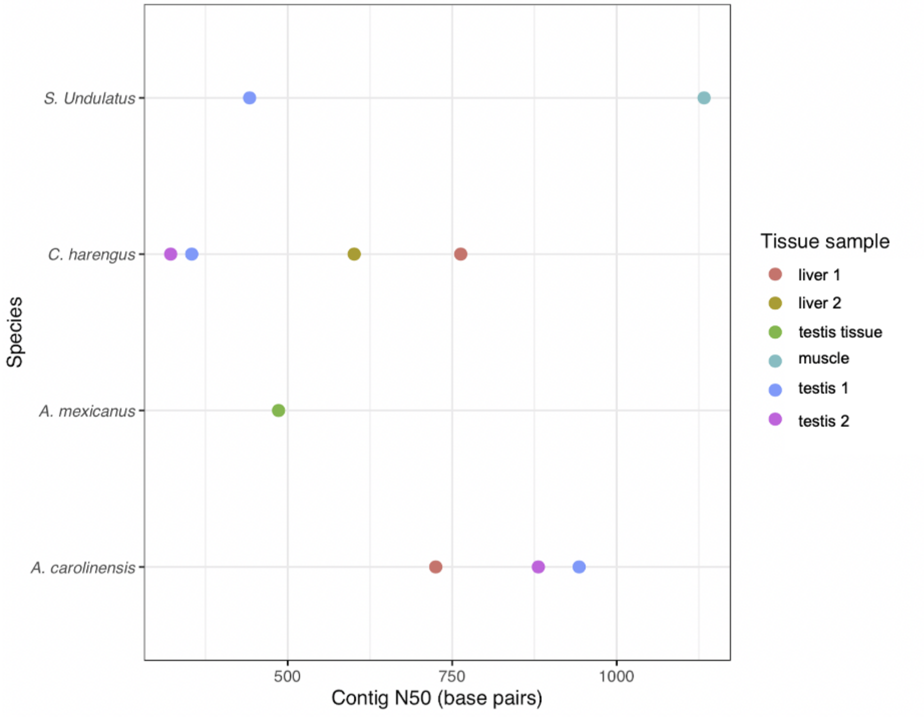

Figure S2. Contig N50 in base pairs as a statistic describing the quality of *de novo* transcriptome assemblies. Colors represent the different tissues used in the two lizard species (*S. undulatus* and *A. carolinensis*) and two fish species (*C. harengus* and *A. mexicanus*).

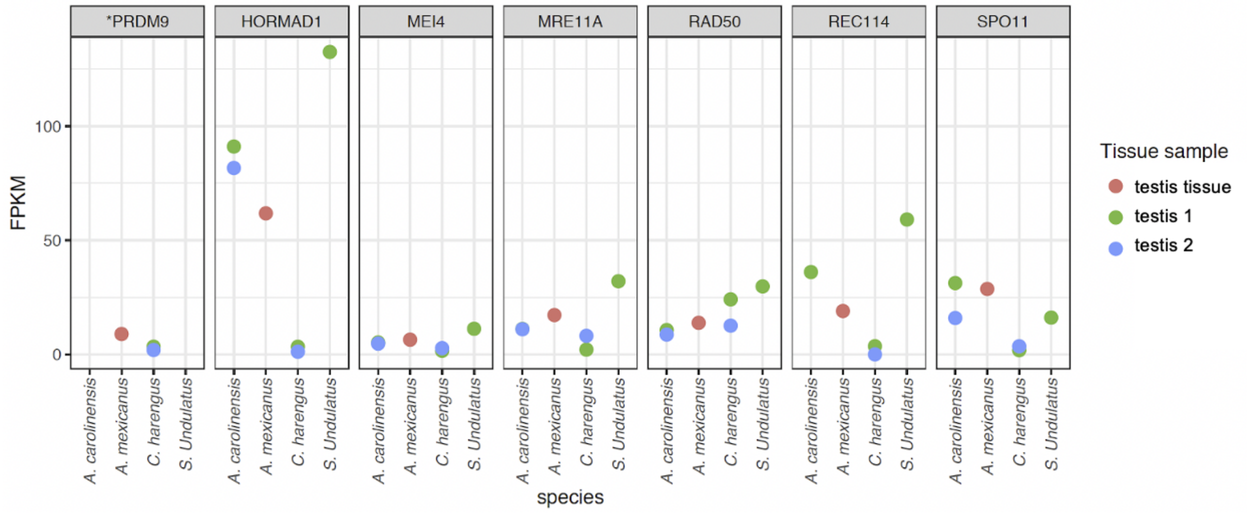

Figure S3. Expression levels of six core meiosis-related genes (26) across species and tissues. The y-axis corresponds to fragments per kilobase of transcript per million mapped reads (FPKM). Despite evidence for expression of the other six core meiotic genes, PRDM9 expression is not detected in *S. undulatus* and *A. carolinensis*.

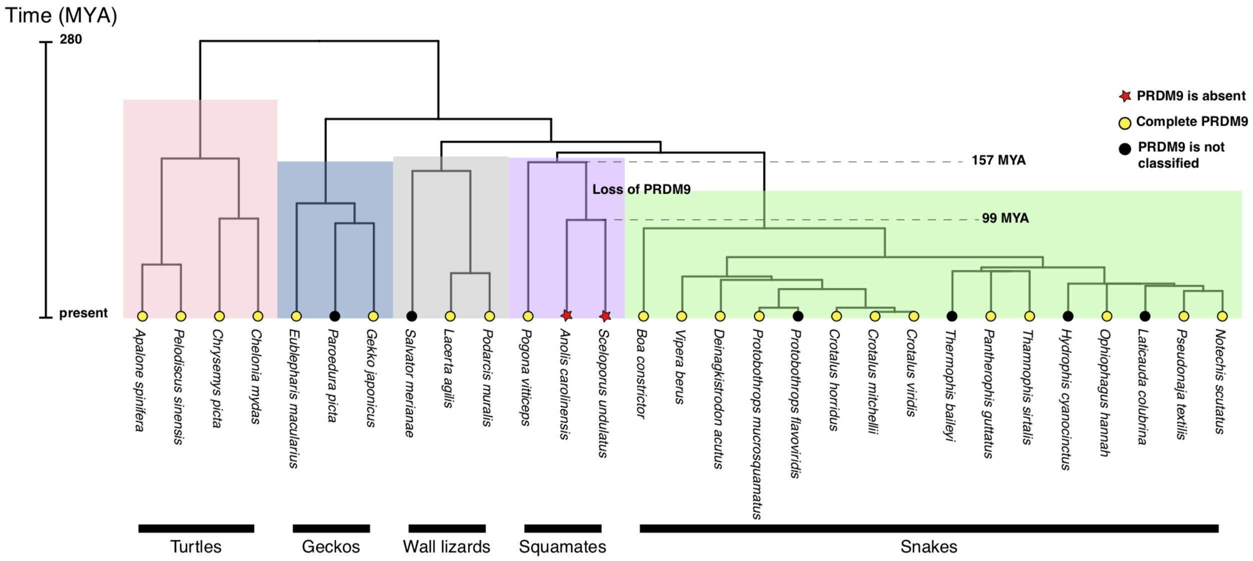

Figure S4. Phylogenetic distribution of *PRDM9* orthologs in reptiles, using the phylogenetic tree and divergence dates obtained from Timetree (11). Species assigned with yellow circles carry a complete *PRDM9*. Species indicated with a red star are ones for which we were unable to identify PRDM9 expression in testis samples. Species indicated with a black circle are species for which Refseq is not available and *PRDM9* classification was therefore not conducted. Based on the phylogenetic relationship between *Anolis carolinensis* and *Sceloporus undulatus*, the *PRDM9* loss shared by these two species likely occurred between 99 and 157 million years ago (mya).

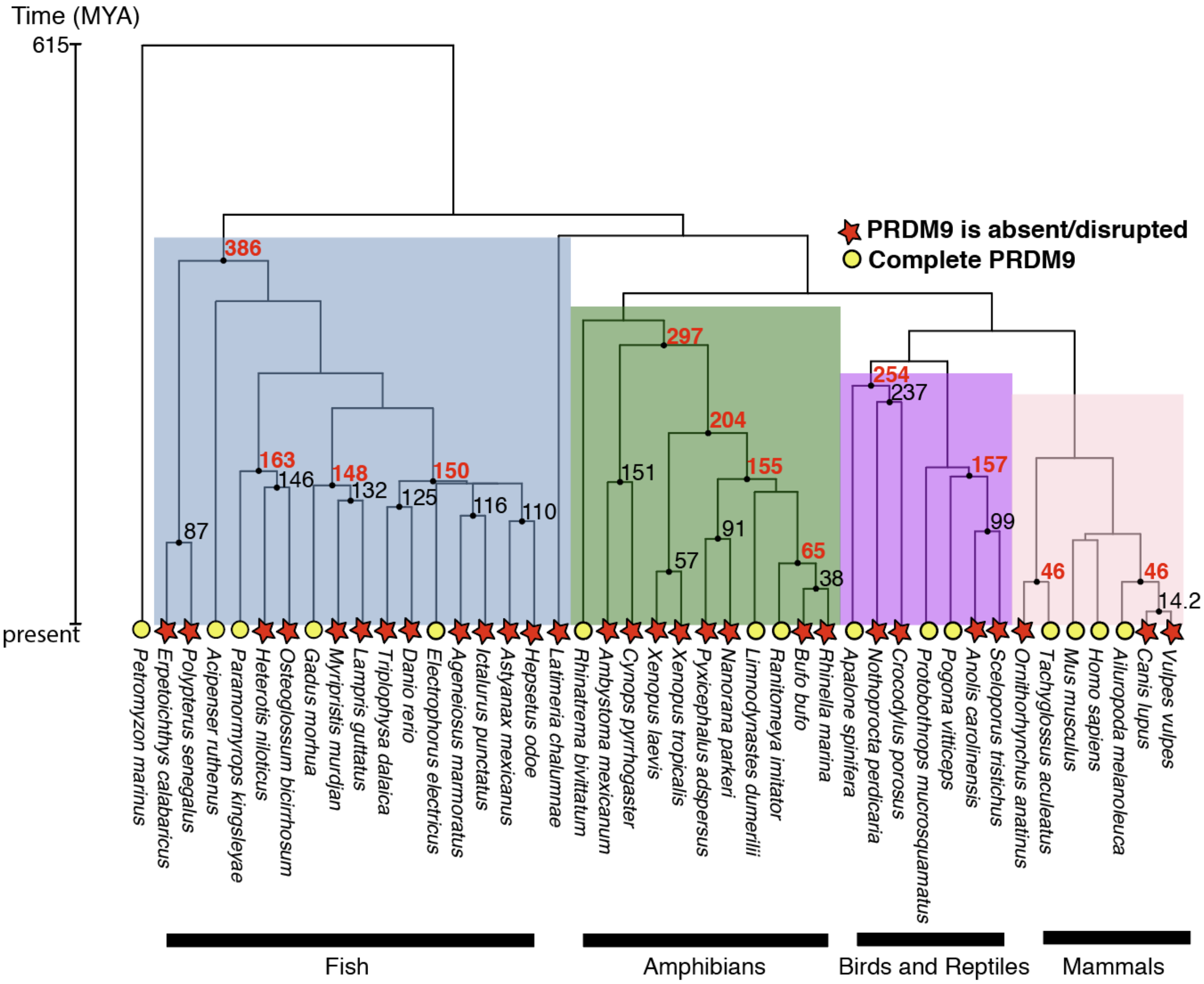

Figure S5. Phylogenetic distribution of *PRDM9* orthologs in vertebrates, using the phylogenetic tree and divergence dates obtained from Timetree (27). A complete *PRDM9* was found in species marked with yellow circles. Species marked with a red star are ones for which we were unable to identify a complete PRDM9. The highlighted dates indicate the inferred timing of the multiple *PRDM9* losses. The 'minimum date' in black reflects the time to the most recent common ancestor amongst species without PRDM9, whereas the 'maximum date' in red is the time to the first common ancestor between species without *PRDM9* and the most closely related species with *PRDM9*. The most recent loss of *PRDM9* occurred either in the branch leading to canids, between 14.2 and 46 million years ago (mya) or potentially the branch leading to platypus (*Ornithorhynchus anatinus*) sometime in the last than 46 mya.

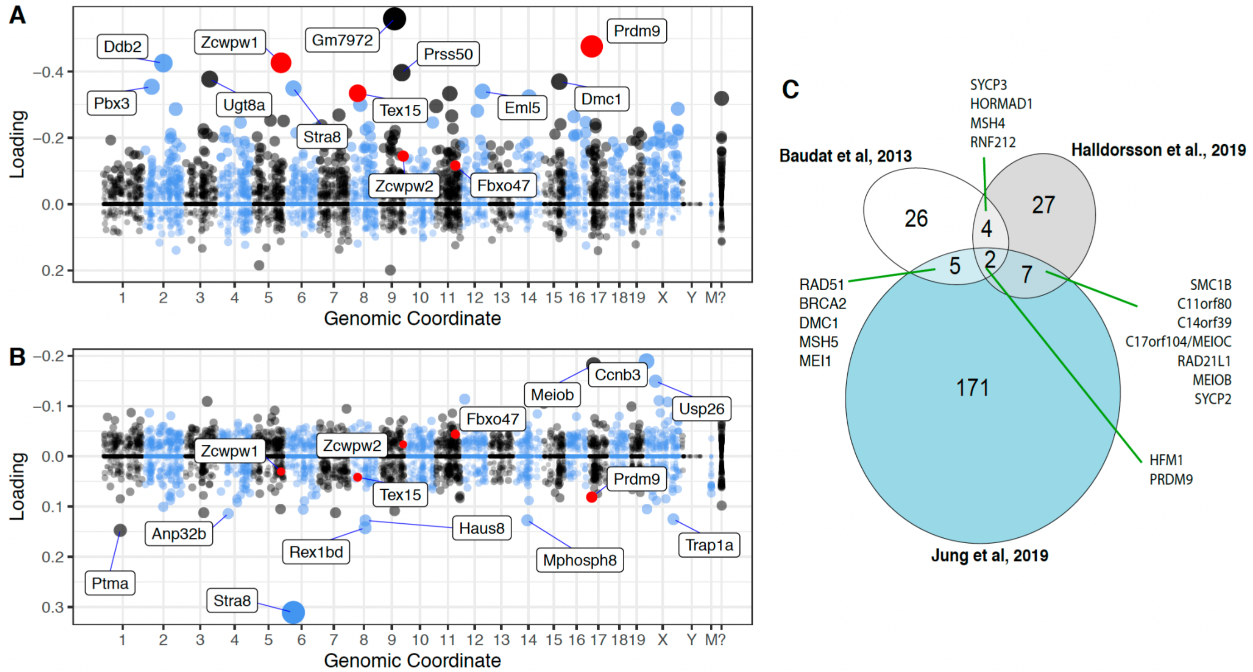

Figure S6. Meiosis-specific candidate genes. (2) inferred 46 principal components from single cell expression patterns during mouse spermatogenesis, which are thought to loosely correspond to regulatory programs. Shown in A-B are the two components in which PRDM9 is most highly expressed. The dot sizes are proportional to the square of the absolute value of the loading, so are indicative of higher expression within each component. PRDM9 and the five genes with p<0.05 in our phylogenetic analysis are shown in red. Mouse genomic coordinates are displayed. (**A**) Component 5 is the one in which PRDM9 has its highest loading; it is associated with double strand break formation and is active during (pre)leptotene (2). (**B**) Component 44 is the component in which PRDM9 has its second highest loading; this component is active during zygotene (2). (**C**) Intersection of candidate genes from three sources: (i) the top 1 percent of genes with highest loadings in component 5 (ii) genes associated with variation in recombination phenotypes in humans (3) and (iii) genes known to have a role in mammalian meiotic recombination from functional studies (as summarized in the review by (4)).

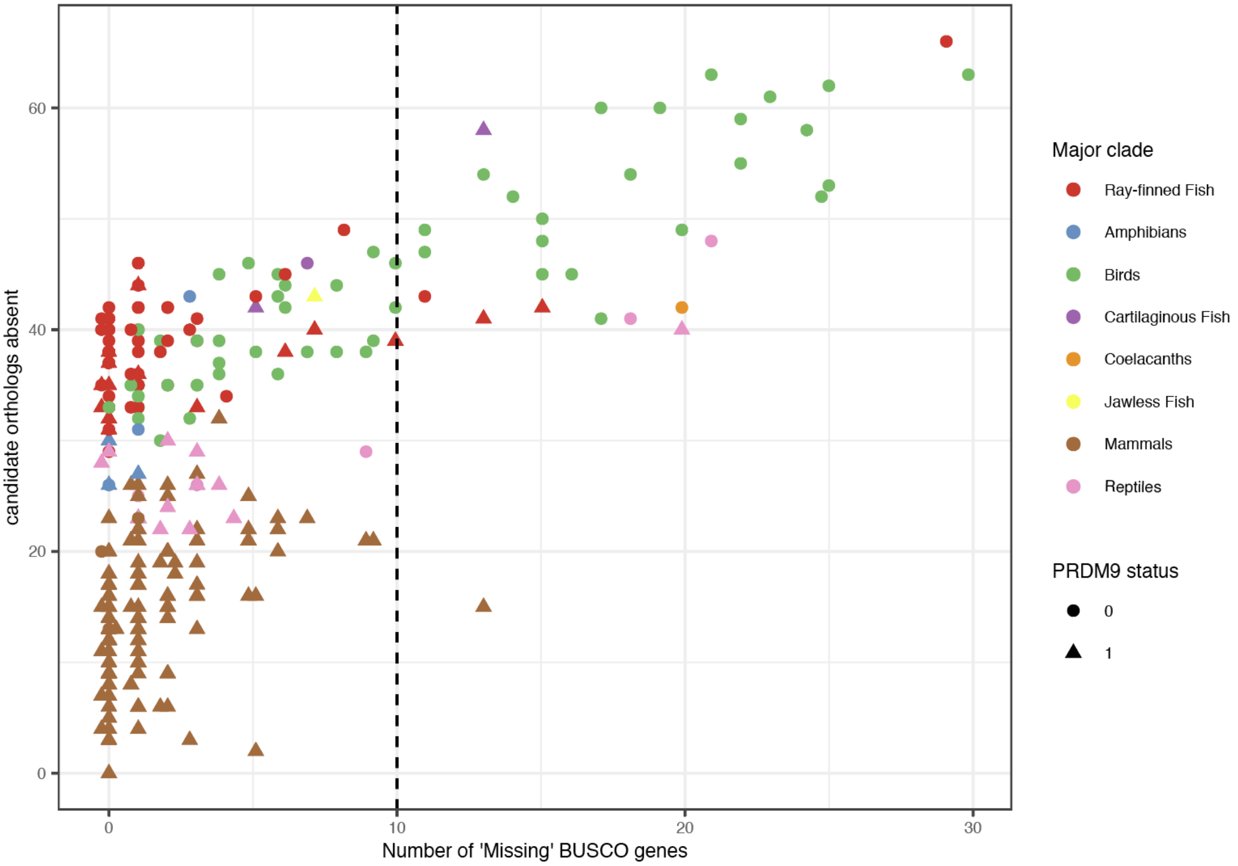

Figure S7. The relationship between the number of candidate genes that were absent in a genome assembly and the number of ‘Missing’ BUSCO genes (28) for that assembly, across species. BUSCO statistics were computed for the most up to date genomes of 339 species (as of November 2020). The relationship is significant (Spearman's rank correlation $\rho = 0.5$, p-value $< 2.2e-16$), suggesting that orthologs of candidate genes of interest might be missed in genomes with low BUSCO scores. In the phylogenetic tests, we therefore considered only species with 10 or fewer missing BUSCO genes (dashed line), leading 32 species to be excluded.

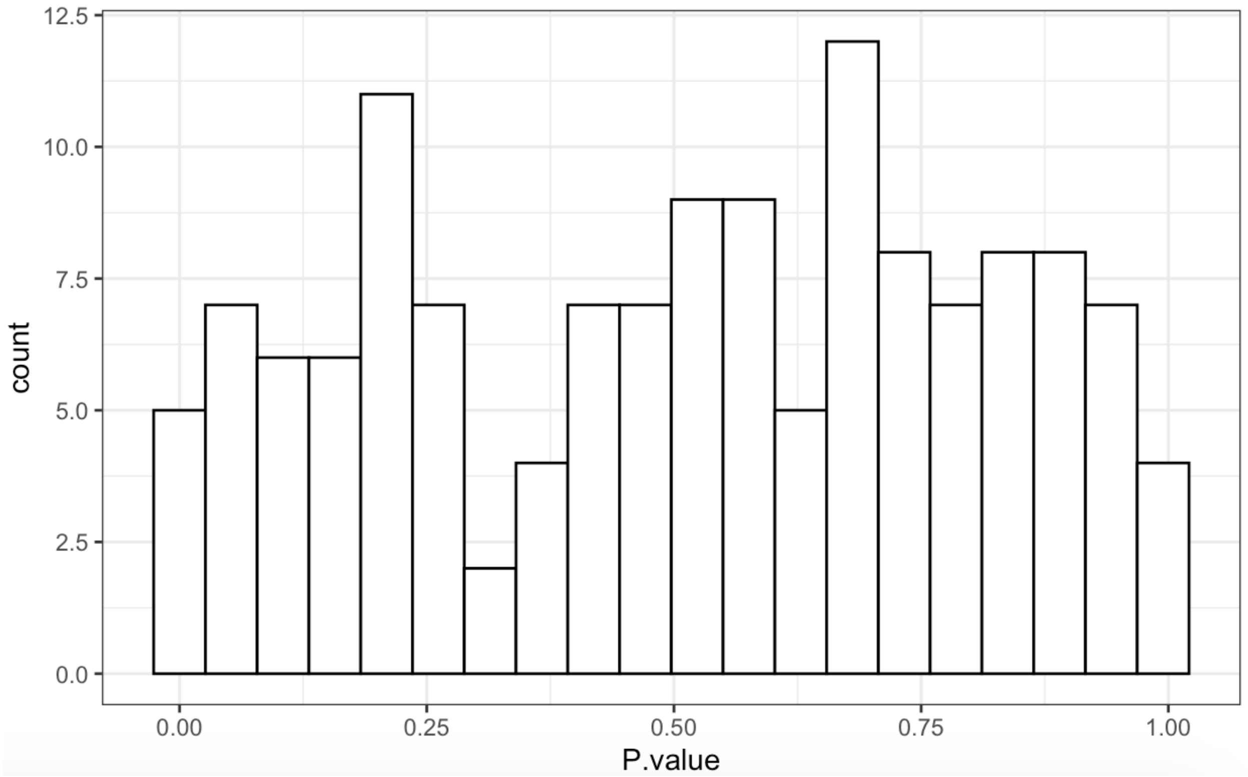

Figure S8. The distribution of p-values obtained across the 139 genes included in phylogenetic tests (individual p-values are available in **Table S7**).

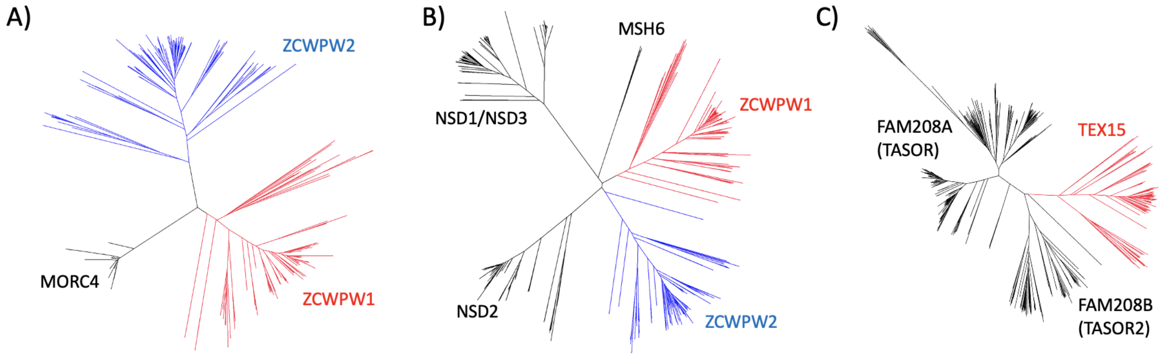

Figure S9. Guide trees created from our initial blastp search results for the zf-CW (**A**) and PWWP (**B**) domains of *ZCWPW1* and *ZCWPW2* orthologs, and the DUF3715 domain of *TEX15* orthologs. Genes were removed if they clustered with *MORC4* in tree **A**, *MSH6*, *NSD1*, *NSD2*, or *NSD3* in tree **B**, *FAM208A* or *FAM208B* in tree **C**. Genes clustering with *ZCWPW1*, *ZCWPW2* or *TEX15* and retained for subsequent analysis are shown in red or blue.

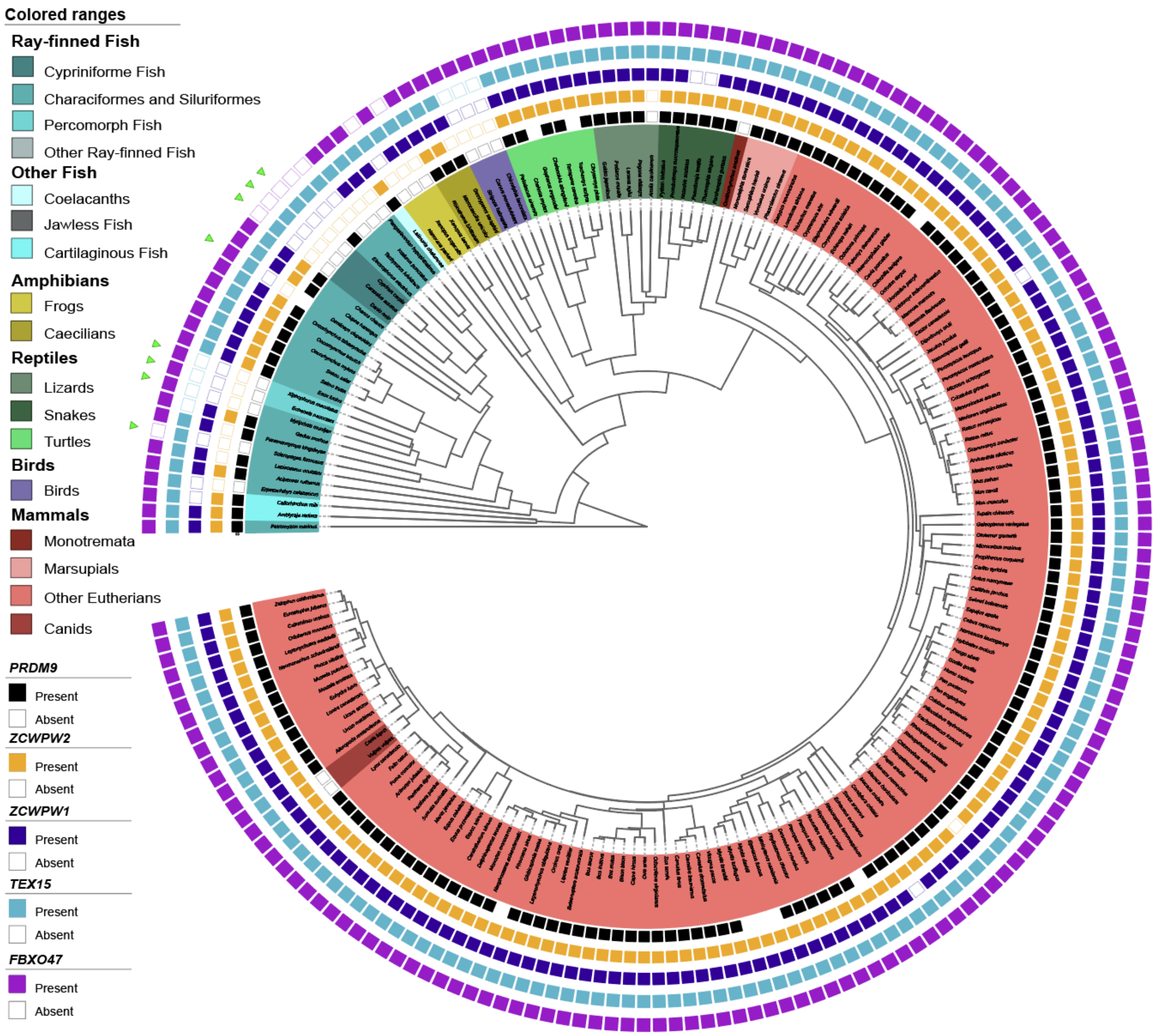

Figure S10. The phylogenetic distribution of *PRDM9* and co-evolving genes across 189 species. Filled teal and empty teal squares indicate whether *PRDM9* is present or absent, respectively (see Methods). If nothing is indicated, the status of *PRDM9* is uncertain. Likewise, filled orange and empty squares indicate whether *ZCWPW2* is present or absent/incomplete; filled and empty navy squares indicate whether *ZCWPW1* is present or absent/incomplete; filled and empty light blue squares indicate whether *TEX15* is present or absent/incomplete; and filled and empty light purple squares indicate whether *FBXO47* is present or absent/incomplete. Green triangles indicate species that only carry *PRDM9* orthologs with substitutions at putatively important catalytic residues in the SET domain (see **Table S4**). The status of candidate genes (for which FDR ≤ 50%; Figure 2A) was re-evaluated based on a search of gene models within whole genome sequences (see Methods); updated p-values for the phylogenetic test are shown in Table 1. The tree was drawn using iTOL (https://itol.embl.de/); an interactive version is available at https://itol.embl.de/shared/izabelcavassim.

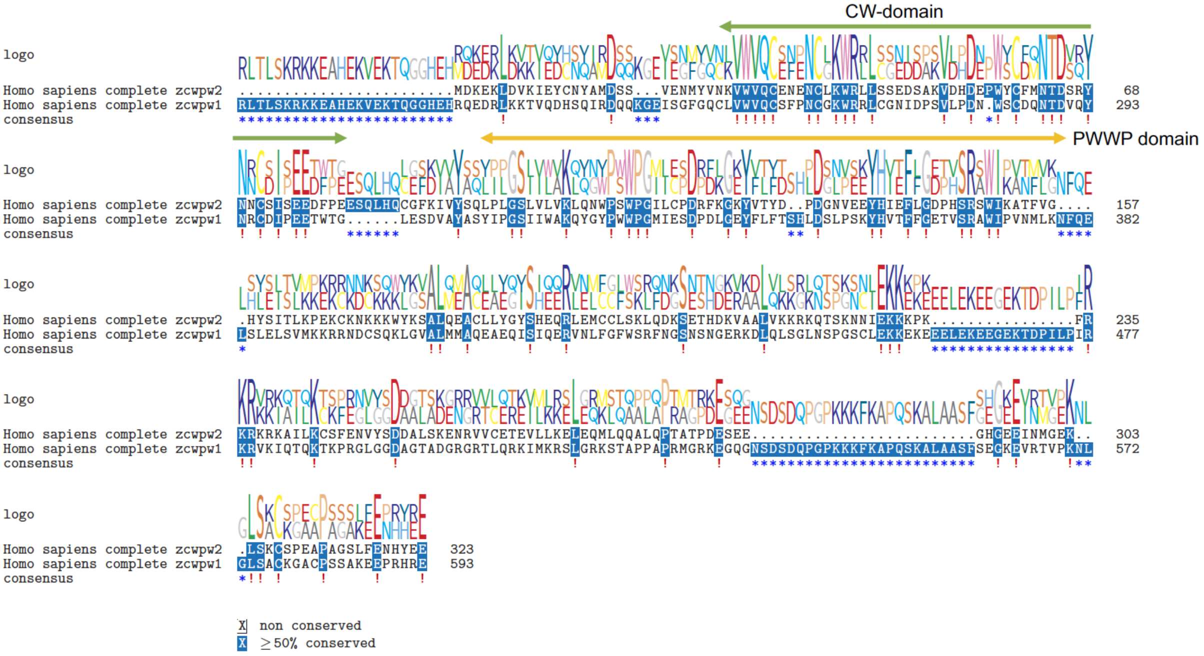

Figure S11: Amino acid sequence alignment between ZCWPW1 and ZCWPW2 proteins from humans. Superfamily domains are marked. In mice, the CW-domain (green arrow) recognizes different methylated states of lysine 4 on histone H3 (H3K4me) (29), while the PWWP domain (yellow arrow) recognizes methylated H3K36 histone tail (30). The SET domain of PRDM9 tri-methylates both histones H3K4 and H3K36 (13).

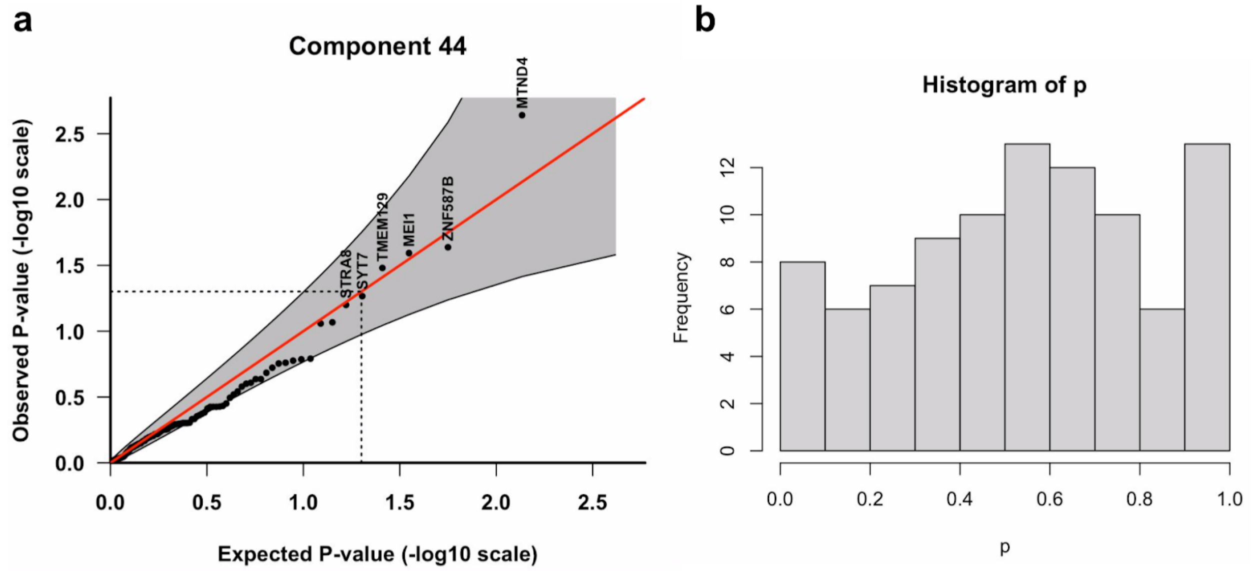

Figure S12. Statistical evidence for the co-evolution of PRDM9 and top 1% of genes

coexpressed with PRDM9 in Component 44 (2). Component 44 is the component in which PRDM9 has its second highest loading in terms of expression; this component is active during zygotene (2). **(A)** Quantile-Quantile plot of the p-values obtained from the phylogenetic tests run on 94 genes that appeared to have been lost at least once in the 189 vertebrate species considered. Genes that are significant at the 5% level are shown in red (outside the dashed lines) and a pointwise 95% confidence interval is shown in grey. **(B)** The distribution of p-values obtained across the 94 genes included in phylogenetic tests.

Supplementary references

1. NCBI Resource Coordinators, Database resources of the National Center for Biotechnology Information. *Nucleic Acids Res.* **46**, D8–D13 (2018).

2. M. Jung, *et al.*, Unified single-cell analysis of testis gene regulation and pathology in five mouse strains. *Elife* **8** (2019).

3. B. V. Halldorsson, *et al.*, Characterizing mutagenic effects of recombination through a sequence-level genetic map. *Science* **363** (2019).

4. F. Baudat, Y. Imai, B. de Massy, Meiotic recombination in mammals: localization and regulation. *Nat. Rev. Genet.* **14**, 794–806 (2013).

5. D. Barker, M. Pagel, Predicting functional gene links from phylogenetic-statistical analyses of whole genomes. *PLoS Comput. Biol.* **1**, e3 (2005).

6. Z. Yang, PAML: a program package for phylogenetic analysis by maximum likelihood. *Bioinformatics* **13**, 555–556 (1997).

7. M. Pagel, Detecting correlated evolution on phylogenies: a general method for the comparative analysis of discrete characters. *Proceedings of the Royal Society of London. Series B: Biological Sciences* **255**, 37–45 (1994).

8. A. Marchler-Bauer, CDD: a Conserved Domain Database for protein classification. *Nucleic Acids Research* **33**, D192–D196 (2004).

9. F. Sievers, D. G. Higgins, Clustal Omega. *Current Protocols in Bioinformatics*, 3.13.1–3.13.16 (2014).

10. E. Birney, M. Clamp, R. Durbin, GeneWise and Genomewise. *Genome Res.* **14**, 988–995 (2004).

11. S. Kumar, G. Stecher, M. Suleski, S. B. Hedges, TimeTree: A Resource for Timelines, Timetrees, and Divergence Times. *Mol. Biol. Evol.* **34**, 1812–1819 (2017).

12. Z. Baker, *et al.*, Repeated losses of PRDM9-directed recombination despite the conservation of PRDM9 across vertebrates. *Elife* **6** (2017).

13. H. Wu, *et al.*, Molecular basis for the regulation of the H3K4 methyltransferase activity of PRDM9. *Cell Rep.* **5**, 13–20 (2013).

14. B. Diagouraga, *et al.*, PRDM9 Methyltransferase Activity Is Essential for Meiotic DNA

Double-Strand Break Formation at Its Binding Sites. *Mol. Cell* **69**, 853–865.e6 (2018).

15. C. P. Ponting, What are the genomic drivers of the rapid evolution of PRDM9? *Trends Genet.* **27**, 165–171 (2011).

16. A. Auton, *et al.*, Genetic recombination is targeted towards gene promoter regions in dogs. *PLoS Genet.* **9**, e1003984 (2013).

17. S. Singhal, *et al.*, Stable recombination hotspots in birds. *Science* **350**, 928–932 (2015).

18. R. Schmieder, R. Edwards, Fast identification and removal of sequence contamination from genomic and metagenomic datasets. *PLoS One* **6**, e17288 (2011).

19. M. G. Grabherr, *et al.*, Full-length transcriptome assembly from RNA-Seq data without a reference genome. *Nat. Biotechnol.* **29**, 644–652 (2011).

20. M. D. Macmanes, On the optimal trimming of high-throughput mRNA sequence data. *Front. Genet.* **5**, 13 (2014).

21. I. Lam, S. Keeney, Mechanism and regulation of meiotic recombination initiation. *Cold Spring Harb. Perspect. Biol.* **7**, a016634 (2014).

22. Applied Research Applied Research Press, *RSEM: Accurate Transcript Quantification from RNA-Seq Data with Or Without a Reference Genome* (2015).

23. B. Langmead, S. L. Salzberg, Fast gapped-read alignment with Bowtie 2. *Nat. Methods* **9**, 357–359 (2012).

24. M. Mahgoub, *et al.*, Dual histone methyl reader ZCWPW1 facilitates repair of meiotic double strand breaks in male mice. *Elife* **9** (2020).

25. D. Wells, *et al.*, ZCWPW1 is recruited to recombination hotspots by PRDM9, and is essential for meiotic double strand break repair. *Elife* **9** (2020).

26. F. Baudat, Y. Imai, B. de Massy, Meiotic recombination in mammals: localization and regulation. *Nat. Rev. Genet.* **14**, 794–806 (2013).

27. S. Kumar, G. Stecher, M. Suleski, S. B. Hedges, TimeTree: A Resource for Timelines, Timetrees, and Divergence Times. *Mol. Biol. Evol.* **34**, 1812–1819 (2017).

28. R. M. Waterhouse, *et al.*, BUSCO Applications from Quality Assessments to Gene Prediction and Phylogenomics. *Mol. Biol. Evol.* **35**, 543–548 (2018).

29. F. He, *et al.*, Complex structure of the zf-CW domain and the H3K4me3 peptide (2010) https:/doi.org/10.2210/pdb2rr4/pdb.

30. S. Qin, J. Min, Structure and function of the nucleosome-binding PWWP domain. *Trends Biochem. Sci.* **39**, 536–547 (2014).
